# Predicting work disability among people with chronic conditions: a prospective cohort study

**DOI:** 10.1038/s41598-023-33120-3

**Published:** 2023-04-18

**Authors:** Solja T. Nyberg, Jaakko Airaksinen, Jaana Pentti, Jenni Ervasti, Markus Jokela, Jussi Vahtera, Marianna Virtanen, Marko Elovainio, G. David Batty, Mika Kivimäki

**Affiliations:** 1 Clinicum, Faculty of Medicine, University of Helsinki, Tukholmankatu 8B, 00014 Helsinki, Finland; 2 Finnish Institute of Occupational Health, Helsinki, Finland; 3 Department of Psychology and Logopedics, Faculty of Medicine, University of Helsinki, Helsinki, Finland; 4 Institute of Criminology and Legal Policy, University of Helsinki, Helsinki, Finland; 5 Department of Public Health, University of Turku, Turku, Finland; 6 Centre for Population Health Research, University of Turku, Turku, Finland; 7 Turku University Hospital, Turku, Finland; 8 School of Educational Sciences and Psychology, University of Eastern Finland, Joensuu, Finland; 9 Division of Insurance Medicine, Karolinska Institutet, Stockholm, Sweden; 10 Finnish Institute for Health and Welfare, Helsinki, Finland; 11 Department of Epidemiology and Public Health, University College London, London, UK

**Keywords:** Risk factors, Epidemiology

## Abstract

Few risk prediction scores are available to identify people at increased risk of work disability, particularly for those with an existing morbidity. We examined the predictive performance of disability risk scores for employees with chronic disease. We used prospective data from 88,521 employed participants (mean age 43.1) in the Finnish Public Sector Study including people with chronic disorders: musculoskeletal disorder, depression, migraine, respiratory disease, hypertension, cancer, coronary heart disease, diabetes, comorbid depression and cardiometabolic disease. A total of 105 predictors were assessed at baseline. During a mean follow-up of 8.6 years, 6836 (7.7%) participants were granted a disability pension. C-statistics for the 8-item Finnish Institute of Occupational Health (FIOH) risk score, comprising age, self-rated health, number of sickness absences, socioeconomic position, number of chronic illnesses, sleep problems, BMI, and smoking at baseline, exceeded 0.72 for all disease groups and was 0.80 (95% CI 0.80–0.81) for participants with musculoskeletal disorders, 0.83 (0.82–0.84) for those with migraine, and 0.82 (0.81–0.83) for individuals with respiratory disease. Predictive performance was not significantly improved in models with re-estimated coefficients or a new set of predictors. These findings suggest that the 8-item FIOH work disability risk score may serve as a scalable screening tool in identifying individuals with increased risk for work disability.

## Introduction

The probability of being in employment is strongly affected by the number of chronic diseases. According to OECD statistics, for example, 1 in 4 people aged 50–59 with no chronic diseases were not in employment, while this was the case for half of those with 2 or more chronic conditions^1^. The number of age-related chronic diseases in working populations is likely to increase in the future due to population ageing. This presents work life with new challenges, including a need for better preventing work disability in working populations which include a growing number of people with a chronic disease.

To enable timely interventions, numerous studies have investigated predictors of work disability in the general and working populations as well as in groups with specific diseases, or those that have undergone specific treatment procedures^2–11^. Fewer studies have examined prediction in high-risk individuals across multiple common chronic conditions that increase the likelihood of work disability. For example, there are no well-validated and easily administered prediction tools available to determine the risk among employees who have depression, musculoskeletal disorders, respiratory disease, hypertension or cardiometabolic multimorbidity. This is an important limitation which may hinder optimal targeting of interventions to those who would benefit most.

The Finnish Institute of Occupational Health (FIOH) has previously formulated a risk prediction model for work disability for use in the general working population^2^. This model had a C-index of 0.8 indicating high discriminative ability. In the present study, we examined whether the variables used also have the capacity to accurately determine work disability risk among employees with chronic conditions. We first evaluated the predictive performance of the 8-item FIOH risk score in nine common disease groups; then developed a modified version where we re-estimated the coefficients; and, lastly, built a model with a new set of predictors selected from a large pool of additional variables and ascertained whether these new models improved risk prediction. To evaluate the relevance of the risk models in clinical decision making, we dichotomized the score to distinguish test positives from test negatives, and examined detection rate and false positive rate for this measure^12^.

## Methods

### Study population

Participants were from the Finnish Public Sector study, a prospective cohort study of public sector employees from 10 municipalities and 21 hospitals in the same geographical areas in Finland. The eligible population represented 31.4% of all municipal employees in Finland at the time of the study, including all employees with job contract in the 10 municipalities and 21 hospitals at the time of the surveys^13^. Participants responded to questionnaire surveys conducted in 2000–2002, 2004, 2008, or 2012. In the present study, we used data from the full study population of respondents including employees with and without chronic conditions. Using self-reports of physician-diagnosed diseases, complemented with records from the cancer registry and drug reimbursement register (for respiratory disease, hypertension, coronary heart disease, musculoskeletal disorders, or diabetes), we categorized participants into subgroups with different chronic conditions including those living with musculoskeletal disorder, depression, migraine, respiratory disease, hypertension, cancer, coronary heart disease, diabetes and comorbid depression and cardiometabolic disease (co-occurrence of mental and physical illnesses with major public health importance) at baseline. We considered the baseline for each participant with disease to be at the survey when the particular condition was first reported. Ethical approval was obtained from the ethics committee of the Helsinki-Uusimaa Hospital District Ethics Committee (HUS/1210/2016). All participants provided a written informed consent. This study was conducted according to the guidelines of the Helsinki declaration. Details of the study design and participants have been previously described^14^.

### Potential risk predictors

We used a pool of 105 variables which included those in the 8-item risk prediction FIOH-model of work disability for the general working population: age category (< 35; 35–39; 40–44; 45–49; 50–54; 55 + years), BMI (< 18.5; 18.5- < 25; 25–30; 30 + kg/m^2^), socioeconomic status (SES), smoking (yes; no), number of chronic diseases (0, 1, 2, 3+), self-rated health (range 1–5), difficulty falling asleep (range from 1 (never) to 6 (almost every night)) and number of sickness absences in previous year before baseline (0, 1, 2, 3+)^2^. Other available variables included sex, alcohol consumption, physical inactivity, psychological distress and working conditions (job control, job demands, job strain, effort, reward and effort-reward imbalance, relational justice, procedural justice, participatory safety, support for innovation, vision, task orientation, social capital at workplace, shift work and working night shifts). We measured SES based on occupational titles categorised according to the occupational classification of Statistics Finland, which is based on the World Health Organization/International Labor Organization International Standard Classification of Occupations (ISCO-88)^15^ into 7 occupational groups: ‘Managers’ (ISCO class 1), ‘professionals’ (ISCO class 2, for example, physicians and teachers), ‘technicians and associate professionals’ (ISCO class 3, for example, registered nurses), ‘clerical support workers’ (ISCO class 4, for example, secretaries), ‘service workers’ (ISCO class 5, for example, cooks), ‘manual workers’ (ISCO classes 6–8, for example, maintenance workers) and ‘elementary occupations’ (ISCO class 9, for example, cleaners).

The predictors are presented in Table 1 and a detailed list of individual items is provided in Appendix (p. 2–4).

**Table 1:** Potential predictors.

Potential predictor	
Demographics (3 items)	Work characteristics (22 items)
Age*	Job strain (scale + 2 items)
Sex	Job control (6 items)
Socioeconomic status*	Job demand (3 items)
	Effort-Reward imbalance (scale + 2 items)
Health (35 items)	Effort (1 item)
BMI*	Reward (3 items)
Jenkins sleep scale (scale + 4 items)*	Shift work
Chronic diseases (sum + 12 items)*	Night shift
Self-rated health*	Social capital at workplace (scale)
No. of sickness absences in previous year*	
GHQ (2 scales + 12 items)	Team climate (18 items)
	Participatory safety (scale + 4 items)
Risk behavior (12 items)	Vision (scale + 4 items)
Smoking*	Task orientation (scale + 3 items)
Alcohol consumption (scale + 5 items)	Support for innovation (scale + 3 items)
Inactivity (scale + 4 items)	
	Management (15 items)
	Relational justice (scale + 6 items)
	Procedural justice (scale + 7 items)

*Items included in the FIOH-model.

### Ascertainment of work disability

All study members were insured in some pension scheme. Records were obtained from the national register at the Finnish Centre of Pensions^16^, an organisation which has a statutory obligation to curate records of all pensions in Finland. Disability pension records including start date and diagnosis according to the World Health Organization International Classification of Diseases, version 10. The outcome was full-time disability pensions (temporary or permanent) which is defined as the capacity for work being impaired by at least 60% due to a disease, injury, or other disability. These records have been widely used in research context^17–19^.

### Statistical methods

We imputed missing data on predictors (3.7% of all observations) as follows: we first complemented missing responses on chronic diseases using data from the cancer registry and the drug reimbursement register, then set responses on chronic diseases that were still missing as ‘no’ answers, and height as median value of all non-missing responses per individual. Other predictors were imputed using single imputation with predictive mean matching^20^.

The follow-up ended in case of death, retirement (age-related or early retirement on other than health grounds), work disability, or a maximum follow-up time of 10 years, whichever came first. The unadjusted bivariate associations between the individual predictor variables and work disability are shown with Manhattan plots. We performed three steps to select the best model for each disease group. In the first, we examined whether the FIOH model was valid within the disease groups. According to the FIOH model, the work disability risk is estimated as follows^2^:

$${\text{P(x) = }}\Phi {\text{[(ln(10) - linear prediction)/scale)]}}$$

where Φ is the standard cumulative normal distribution. We applied the model and the coefficients that were formulated by Airaksinen et al.^2^. Coefficients for linear predictor in the FIOH-model were fitted with lognormal distribution and are provided in the report by Airaksinen et al.^2^ and in the Appendix of this paper (page 5).

The second step was to examine whether the model for each disease groups could be improved by re-estimating the coefficients of the FIOH-model, using the same set of the explanatory variables as was used in the FIOH model. We call this the re-estimated FIOH model. For fitting the models, we used a similar method (including assumption of lognormal distribution) as Airaksinen et al.^2^.

The third step was to examine whether the prediction could be improved by selecting a completely new set of predictors for each disease group. Following the conventional approach in developing prediction algorithms for survival data, we fitted the models with Weibull distribution^21,22^. For each disease group we first ran a redundancy analysis to exclude variables that could be readily predicted using all other variables. We then specified a parametric survival model that included all the candidate variables as predictors (‘full model’). To obtain a more parsimonious algorithm, we derived the predicted work disability risks from the full model for each individual. We then used backward stepwise ordinary least squares regression to select eight predictors, by predicting risks derived from the full model as the outcome. The number of items in the new models was chosen to be the same as in the FIOH-model because a short questionnaire is easy to administer and quick to respond, maximising the response rate. If the selected eight predictors included any summary variables (for example job strain), we repeated the previous steps with the summary variable(s) broken down to individual items. The models achieved for each disease group as a result of the third step are referred as the new model.

The performance of each prediction model was evaluated using Harrell’s C-index, which is the concordance between predicted and observed survival^20^. This index gives the probability that a randomly selected individual who experienced the outcome during the follow-up, had a higher risk score than a randomly selected individual who did not experience the outcome. The C-index has a range from 0.5 (no predictive ability) to 1 (maximum predictive ability). C-index under 0.7 represents poor, 0.7–0.8 good, and > 0.8 strong discrimination ability. To examine whether the original findings from the general working population were replicable in our dataset, we computed C-statistics for the entire study population with and without chronic conditions and among those with no history of sickness absence one year prior to baseline (the low-risk population^11^). Calibration of the model—that is, how accurately the predicted absolute risks correspond to the observed absolute risks—was assessed using calibration plots. We additionally plotted observed and predicted events by deciles of 10-year risk for each model, excluding by age and baseline those with less than 10 years of potential follow-up time. We compared the performance of the FIOH-model with both new models using the C-statistics with 95% confidence intervals. If the confidence intervals were overlapping, the FIOH-model was chosen.

To evaluate the final model for an individual, we dichotomized the score into ‘test positive’ versus ‘test negative’ using alternative thresholds of 5; 10; 20; 30; 40; 50 and 60% for a positive test result. For positive test cases, we calculated false positive rate (the proportion of test positive cases who did not experience work disability), detection rate (the proportion of work disability cases who were test positive) and the ratio of true to false positives. The formulas were as follows:

$$\begin{aligned} & {\text{False}}\;{\text{positive}}\;{\text{rate}} = {\text{b}}/\left( {{\text{b}} + {\text{d}}} \right) \\ & {\text{Detection}}\;{\text{rate}} = {\text{a}}/\left( {{\text{a}} + {\text{c}}} \right) \\ \end{aligned}$$

Ratio of true to false positives = 1 : (b/a), where a, b, c and d represent different combinations of risk scores and work disability as defined below:

**Table:** 

Risk score	Work disability during the follow-up	
	Yes	No
Test positive	a	b
Test negative	c	d

All analyses were performed using R 4.2.2 (packages: mice^23^, rms^24^, leaps^25^ and Hmisc^26^) and SAS 9.4.

### Ethics approval

Ethical approval was obtained from the ethics committee of the Helsinki-Uusimaa Hospital District Ethics Committee (HUS/1210/2016).

### Consent to participate

Informed consent was obtained from all individual participants included in the study.

## Results

### Baseline characteristics

**Figure 1:**
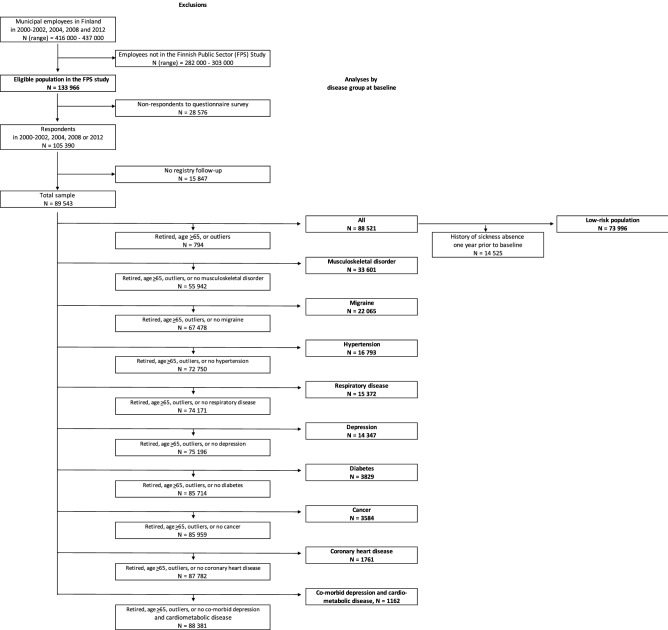
Flow diagram for total sample and disease groups.

Figure 1 shows sample selection. The target population was municipal employees during the survey years in Finland, on average of 426,500 men and women during the survey years. The eligible population in the 10 towns and 21 hospitals which participated in the FPS study represented 31.4% of all municipal employees. Of these, 78.7% responded to at least one of the four questionnaire surveys. Linked records from national health and work disability registers were available for 85.0% of the respondents, a total of 89 543 adults. We excluded from analyses people who were on disability pension or retired, at age 65 years or older or with extreme values in BMI (< 15 or > 50) at baseline.

Table 2 shows characteristics of the resulting analytical sample of 88,521 participants. Of them, 70,805 were women, the mean age was 43.1 years, and the largest occupational groups were ‘professionals’ (for example, teachers) and ‘associate professionals’ (for example, registered nurses). 73,996 had no history of short-term work disability (no sickness absences one year before baseline) and this group was denoted as the low-risk population. The number of participants with specific, prevalent condition at baseline varied between 1162 (comorbid depression and cardiometabolic disease) and 33,601 (musculoskeletal disorders).

**Table 2:** Baseline characteristics of all participants and subgroups of individuals with no history of sickness absence and those with a chronic condition at baseline.

Baseline characteristic	All*	Low risk population†	MSD	Migraine	Hypertension	Respiratory disease	Depression	Diabetes	Cancer	CHD	Depression & CMD
	(N = 88,521)	(N = 73,996)	(N = 33,601)	(N = 22,065)	(N = 16,793)	(N = 15,372)	(N = 14,347)	(N = 3829)	(N = 3584)	(N = 1761)	(N = 1162)
Sex											
Women	80.0	79.2	81.2	89.9	76.5	82.5	83.9	72.2	88.1	63.5	74.0
Men	20.0	20.8	18.8	10.1	23.5	17.5	16.1	27.8	11.9	36.5	26.0
Age, y											
< 35	22.3	23.7	7.2	21.0	3.2	16.8	12.6	8.3	4.2	2.5	6.0
35–39	14.5	15.0	9.0	14.9	5.3	12.8	12.4	8.1	5.9	2.7	6.6
40–44	16.5	16.6	14.1	16.8	11.2	16.1	16.5	11.5	10.2	4.9	9.7
45–49	16.4	16.1	19.0	16.3	17.4	16.9	19.4	14.6	16.6	11.5	15.8
50–54	16.0	15.2	23.2	16.3	27.1	17.7	19.5	21.6	25.0	25.3	25.7
55 +	14.4	13.4	27.6	14.7	35.9	19.8	19.7	36.1	38.2	53.1	36.2
BMI, kg/m^2^											
< 18.5	1.3	1.3	0.7	1.5	0.4	1.2	1.1	0.4	0.7	0.6	0.4
18.5– < 25	54.5	56.1	44.6	53.6	29.9	46.6	48.2	22.8	48.8	35.0	28.7
25–30	31.8	31.2	37.1	31.1	41.4	34.1	33.6	35.4	35.6	40.9	35.3
30 +	12.5	11.4	17.6	13.9	28.4	18.2	17.0	41.4	14.9	23.5	35.6
Socio-economic status											
Managers	2.2	2.4	2.8	2.2	3.5	2.7	2.3	3.3	3.2	3.8	2.4
Professionals	28.9	30.9	25.7	29.1	24.9	30.4	30.2	24.4	30.9	21.1	24.3
Technicians and associate professionals	27.1	27.4	22.7	26.5	23.1	23.7	24.1	23.2	23.8	21.0	22.9
Clerical support workers	6.4	6.4	7.1	7.5	8.3	7.5	7.9	8.3	8.9	8.3	9.9
Service workers	21.5	20.1	25.1	23.0	22.4	22.5	22.5	21.0	21.5	19.8	21.3
Manual workers	4.2	4.0	5.2	2.7	6.1	3.9	3.6	7.3	3.0	10.3	8.3
Elementary occupations	9.7	8.8	11.4	8.9	11.6	9.1	9.5	12.5	8.8	15.8	11.0
Smoking											
No	81.0	81.8	80.1	82.9	82.5	79.6	76.6	78.8	85.3	81.2	75.8
Yes	19.0	18.2	19.9	17.1	17.5	20.4	23.4	21.2	14.7	18.8	24.2
Chronic illness ‡											
0	64.6	67.3	53.8	0.0	50.9	23.7	0.0	0.0	59.0	0.0	0.0
1	27.7	26.4	33.1	70.4	33.8	47.1	56.9	57.1	28.8	31.8	0.0
2	6.6	5.5	10.5	24.7	11.3	22.7	34.7	27.9	8.2	36.5	46.6
3+	1.2	0.9	2.6	4.9	4.0	6.5	8.4	15.0	4.0	31.7	53.4
Self-rated health											
1 (highest)	41.5	45.3	22.1	32.9	17.0	25.8	19.0	14.5	22.7	9.9	10.1
2	35.3	35.5	38.3	37.6	37.3	36.7	35.2	34.5	37.8	27.3	23.1
3	19.1	16.8	31.1	23.6	35.5	28.9	33.2	37.2	29.8	40.9	37.6
4	3.7	2.3	7.8	5.4	9.2	7.8	11.3	12.5	8.4	18.8	25.4
5 (lowest)	0.3	0.2	0.7	0.6	0.9	0.8	1.3	1.3	1.3	3.1	3.9
Difficulty falling asleep											
1 (never)	46.2	47.8	40.5	41.1	41.0	39.5	29.7	44.0	42.1	38.4	29.9
2	28.2	28.8	26.9	27.8	26.0	27.3	24.6	24.9	26.7	23.1	20.6
3	12.7	12.4	14.4	14.2	14.4	15.0	16.3	13.3	12.9	15.8	16.0
4	9.0	8.0	11.8	11.5	11.6	11.8	17.2	11.7	11.7	12.8	17.0
5	1.5	1.2	2.2	1.9	2.2	2.3	4.1	1.9	2.4	3.2	4.7
6 (almost every night)	2.5	1.8	4.2	3.6	4.8	4.2	8.0	4.3	4.3	6.8	12.0
Number of sickness absences in previous year											
0	83.6	100.0	74.3	79.6	76.2	76.7	66.7	76.2	64.6	67.0	64.0
1	13.4	0.0	19.9	16.3	18.2	18.1	25.4	18.1	28.6	23.9	24.6
2	2.5	0.0	4.8	3.5	4.6	4.2	6.5	4.7	6.0	7.0	8.3
3+	0.5	0.0	1.1	0.7	1.1	1.0	1.4	1.1	0.8	2.2	3.1

CHD, coronary heart disease; CMD﻿, cardiometabolic disease (diabetes or CHD); MSD, musculoskeletal disorder.

*Participants with and without chronic conditions at baseline.

^†^Participants with no sickness absence at baseline.

^‡^Self-reported bronchial asthma, myocardial infarction, angina pectoris, cerebrovascular diseases, migraine, depression or diabetes.

### Work disability during follow-up

**Figure 2:**
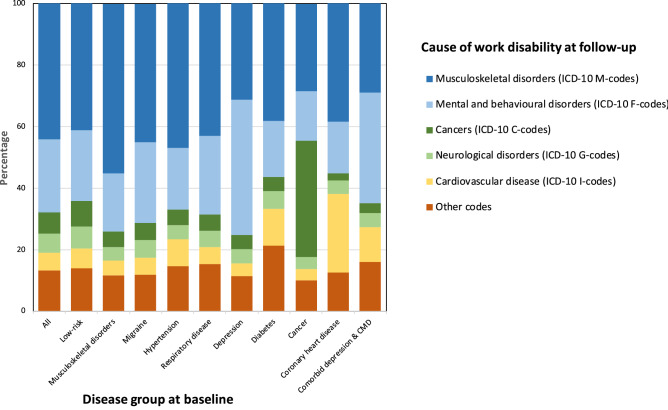
Causes of work disability at follow-up in all participants and those with prevalent diseases.

During a mean follow-up of 8.6 years, 6836 (7.7%) participants were granted a disability pension. The incidence of work disability was 14.1% in those with musculoskeletal disorders, 9.9% for migraine, 15.1% for hypertension, 12.2% for respiratory disease (chronic bronchitis or asthma), 16.7% for depression, 16.4% for diabetes, 14.2% for cancer, 22.6% for coronary heart disease, and 27.7% for comorbid depression and cardiometabolic disease. The most common causes of work disability were diseases of the musculoskeletal system and connective tissue (44.2%), followed by mental, behavioural and neurodevelopmental disorders (23.7%), and these proportions varied by disease group (Fig. 2, for details, see Appendix p. 6).

### Associations of potential risk predictors and work disability

**Figure 3:**
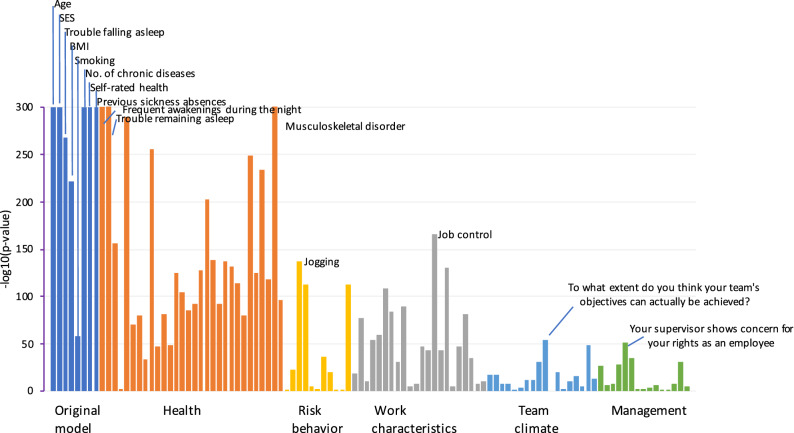
Bivariate unadjusted associations between predictors at baseline and work disability at follow-up in all participants with and without chronic disease at baseline. Bars represent -LOG10(p-value) and are cut at the maximum value of 300.

The unadjusted bivariate associations between 105 predictor variables and work disability are shown in Fig. 3. All variables of the 8-item FIOH-risk score were associated with work disability (*p* < 0.0001), the strongest associations seen for age and self-rated health. Many other health-related variables were also strongly associated with work disability whereas the associations of the items related to risk behaviours, work characteristics, team climate and management were weaker. The pattern of findings was similar in all disease groups (Appendix, p. 7–15).

### Selection of the model for each disease group

Development of the re-estimated and new models is reported in Appendix (p. 16–17.) The predictors in the new models included 4 to 7 of the 8 predictors in the FIOH-model, depending on the participant group. Each of these models included age, socioeconomic status, self-rated health and the number of sickness absences in previous year. Individual chronic diseases were also part of the models for every disease group. Work-related items were included in the models for musculoskeletal disorders, hypertension, respiratory disease, diabetes, cancer, and comorbid depression and cardiometabolic disease.

**Figure 4:**
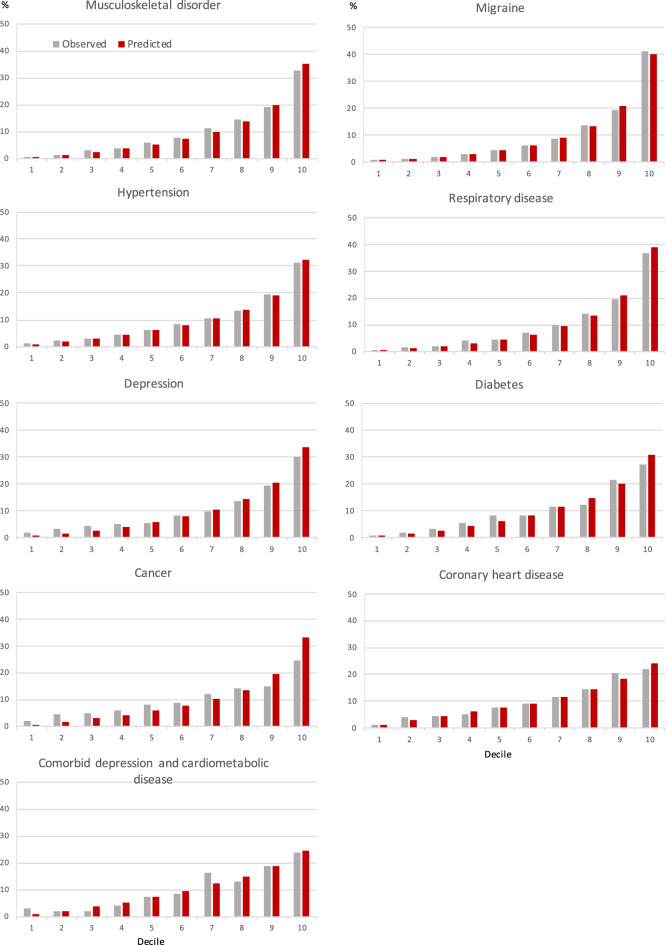
Observed and predicted incidence of work disability by deciles of the work disability risk score.

Table 3 shows that C-statistics for the FIOH-model among all employees, that is, those with and without chronic conditions was (0.84, 95% CI 0.84 to 0.85), a similar C-statistics in the magnitude as in the original study^2^. This result was little changed in analysis of complete case without imputations (N = 78 479, C-statistics 0.84, 95% CI 0.84 to 0.85). Among those with no sickness absence at baseline the C-statistic was (0.82, 95% CI 0.81 to 0.83). The table also provides a comparison of the performance between the FIOH-model and the two alternatives. The FIOH-model performed well in all disease groups. The C-statistics was ≥ 0.80 in those with musculoskeletal disorders (0.80, 95% CI 0.80 to 0.81), migraine (0.83, 95% CI 0.82 to 0.84) and respiratory disease (0.82, 95% CI 0.81 to 0.83). For all other subgroups, including hypertension, depression, diabetes, cancer, coronary heart disease or comorbid depression and cardiometabolic disease, C-statistics was ≥ 0.72 but less than 0.80. The C-statistics for the re-estimated FIOH-models and the new models were virtually the same as for the FIOH-model, suggesting no improvement in predictive performance. Calibration plots for the existing model indicated a high correspondence between the predicted and the observed risk in all disease groups (Fig. 4).

**Table 3:** C-index for the FIOH, re-estimated and new prediction models in all participants and subgroups of individuals with no history of sickness absence and those with a chronic condition at baseline.

Population	C-index (95% confidence intervals)		
	FIOH model	Re-estimated FIOH model	New model
All*	0.84 (0.84, 0.85)		
Low risk population†	0.82 (0.81, 0.83)		
Disease group at baseline			
Musculoskeletal disease	0.80 (0.80, 0.81)	0.80 (0.80, 0.81)	0.80 (0.79, 0.81)
Migraine	0.83 (0.82, 0.84)	0.83 (0.83, 0.84)	0.83 (0.83, 0.84)
Hypertension	0.79 (0.78, 0.80)	0.79 (0.78, 0.80)	0.79 (0.79, 0.80)
Respiratory disease	0.82 (0.81, 0.83)	0.82 (0.81, 0.83)	0.82 (0.81, 0.83)
Depression	0.78 (0.77, 0.78)	0.78 (0.77, 0.79)	0.78 (0.77, 0.78)
Diabetes	0.78 (0.76, 0.80)	0.79 (0.77, 0.81)	0.79 (0.77, 0.81)
Cancer	0.72 (0.70, 0.74)	0.74 (0.71, 0.76)	0.74 (0.72, 0.76)
Coronary heart disease	0.76 (0.73, 0.78)	0.76 (0.74, 0.78)	0.77 (0.74, 0.79)
Comorbid depression and CMD^‡^	0.77 (0.74, 0.79)	0.78 (0.76, 0.80)	0.78 (0.75, 0.80)

*Participants with and without chronic conditions at baseline.

^†^Participants with no sickness absence at baseline.

^‡^CMD, cardiometabolic disease (diabetes or coronary heart disease).

### Predictive performance of the FIOH model

Table 4 shows the detection and false positive rates for dichotomized FIOH-risk scores using various risk thresholds to dichotomise the score to indicate high versus low risk (for results of sensitivity, specificity and positive and negative predictive value, see Appendix, p. 18). With a high threshold for risk (≥ 50% predicted probability indicating high risk), the false positive rate ranged between 2.6% and 8.6% in all disease groups. Exceptions were participants with coronary heart disease (19.0%) and those with comorbid depression and cardiometabolic disease (20.1%) for whom false positive rate was markedly higher. The detection rate varied between 18.9% (participants with cancer) and 52.8% (participants with comorbid depression and cardiometabolic disease) and the ratio of true-to-false positives ranged from 1:1.0 to 1:1.4. With a low threshold (5% predicted probability indicating high risk), detection rate raised to 92.0% or 99.2% (less than 1 in 10 disability cases were missed), but with very high false positive rate (54.2% to 94.4% depending on the disease group). For both the 50% and 5% thresholds, the detection and false positive rates were slightly lower in the total working population, but the ratio of true-to-false positives was approximately the same as in the population with chronic diseases.

**Table 4:** Detection rate, false positive rate and the ratio true-to-false positives for the FIOH prediction model in all participants and subgroups of individuals with no history of sickness absence and those with a chronic condition at baseline.

Predictive performance for a positive test	Threshold for a positive test result						
	5	10	20	30	40	50	60
Musculoskeletal disorders							
Detection rate (%)	95.3	86.6	65.9	46.6	32.7	21.1	12.8
False positive rate (%)	70.2	50.4	26.6	13.6	7.1	3.6	1.7
Ratio true to false positives	1:4.5	1:3.5	1:2.5	1:1.8	1:1.3	1:1.0	1:0.8
Migraine							
Detection rate (%)	92.0	83.1	63.6	47.6	33.3	22.1	14.3
False positive rate (%)	54.2	37.0	19.3	10.0	5.2	2.6	1.2
Ratio true to false positives	1:5.4	1:4.1	1:2.8	1:1.9	1:1.4	1:1.1	1:0.7
Hypertension							
Detection rate (%)	96.4	89.6	71.2	53.2	37.3	24.8	15.2
False positive rate (%)	80.5	61.7	34.4	17.9	9.4	4.9	2.2
Ratio true to false positives	1:4.7	1:3.9	1:2.7	1:1.9	1:1.4	1:1.1	1:0.8
Respiratory disease							
Detection rate (%)	93.0	84.8	66.0	49.9	35.8	24.1	15.1
False positive rate (%)	59.9	42.9	23.2	12.9	7.2	3.8	1.8
Ratio true to false positives	1:4.6	1:3.6	1:2.5	1:1.9	1:1.4	1:1.1	1:0.8
Depression							
Detection rate (%)	93.4	86.2	69.7	53.7	40.1	27.4	18.0
False positive rate (%)	72.3	54.3	31.7	18.5	10.7	6.1	3.0
Ratio true to false positives	1:3.9	1:3.2	1:2.3	1:1.7	1:1.3	1:1.1	1:0.8
Diabetes							
Detection rate (%)	97.0	91.6	77.7	62.4	46.3	32.8	20.7
False positive rate (%)	80.8	68.0	46.3	27.6	16.1	8.6	4.3
Ratio true to false positives	1:4.2	1:3.8	1:3.0	1:2.3	1:1.8	1:1.3	1:1.1
Cancer							
Detection rate (%)	93.1	80.4	58.3	42.2	30.3	18.9	13.2
False positive rate (%)	78.8	59.3	33.5	18.8	9.7	4.3	2.1
Ratio true to false positives	1:5.1	1:4.5	1:3.5	1:2.7	1:1.9	1:1.4	1:1.0
Coronary heart disease							
Detection rate (%)	99.2	97.2	88.9	76.1	62.8	47.2	31.7
False positive rate (%)	94.4	87.5	66.4	45.5	30.3	19.0	10.3
Ratio true to false positives	1:3.3	1:3.1	1:2.6	1:2.0	1:1.7	1:1.4	1:1.1
Comorbid depression and cardiometabolic disease							
Detection rate (%)	98.1	96.3	90.7	79.8	67.7	52.8	38.5
False positive rate (%)	89.0	79.9	61.9	45.2	32.3	20.1	11.8
Ratio true to false positives	1:2.4	1:2.2	1:1.8	1:1.5	1:1.2	1:1.0	1:0.8
Low-risk population							
Detection rate (%)	81.6	63.5	35.0	17.2	8.3	3.5	1.2
False positive rate (%)	38.2	21.5	7.7	2.7	0.9	0.4	0.1
Ratio true to false positives	1:8.1	1:5.9	1:3.8	1:2.7	1:2.0	1:1.9	1:1.6
Total cohort							
Detection rate (%)	87.9	75.0	52.5	35.9	24.0	15.0	9.1
False positive rate (%)	43.7	27.0	12.0	5.7	2.8	1.4	0.62
Ratio true to false positives	1:5.9	1:4.3	1:2.7	1:1.9	1:1.4	1:1.1	1:0.8

## Discussion

Our study shows that a short, self-administered survey instrument has predictive utility for work disability in people with chronic conditions and comorbidity. This survey-based 8-item risk calculator includes age, self-rated health, number of sickness absences, socioeconomic position, the number of comorbidities, sleep problems, body mass index, and smoking habit. The algorithm can be used in many settings, including members of the public who have web access, and the assessment does not require laboratory testing or other clinical measurements. The calculator might be used to identify working-age people with common chronic diseases with an elevated risk of future work disability and so might facilitate early intervention.

Approximately one third of people at age 40 have a chronic condition and the proportion rises to 75% by age 65^27^. Despite this high prevalence and the urgent need for new measures to prevent their work disability, we are not aware of other large-scale studies on risk stratification algorithms for work disability in employees with chronic conditions. In our study, C-index exceeded 0.72 in all disease groups and was 0.80 or greater for those with musculoskeletal disorders, migraine and respiratory disease. These results indicate good discrimination and are comparable to those reported for established risk prediction tools currently used in clinical practice. For example, the C-index is 0.72 for the Pooled Cohort Equations to predict the 10-year risk of cardiovascular disease events using 9 risk factors (age, sex, race, diabetes status, smoking status, antihypertensive medication use, total cholesterol, HDL cholesterol levels, and systolic blood pressure)^28^; between 0.74 and 0.77 for the FINDRISC model to predict the risk of type 2 diabetes using 8 characteristics (age, BMI, waist circumference, physical activity, diet, history of antihypertensive medication use, history of high blood glucose and family history)^29,30^; and 0.70 to 0.91 for QRISK3 to predict future cardiovascular disease based on a wide range of risk factors obtained from electronic health records (age, sex, ethnicity, socioeconomic deprivation, angina or heart attack in a 1st degree relative at age < 60, chronic kidney disease, migraine, corticosteroids, systemic lupus erythematosus, use of atypical antipsychotics, severe mental illness, steroid treatment, erectile dysfunction, total/ HDL cholesterol ratio, BMI, systolic blood pressure variability)^31^.

In clinical decision making, dichotomised predictive scores are used to distinguish patients who should receive intervention or referrals for further assessments, although few studies have reported relevant performance metrics in this regard^12^. While the performance of our dichotomized work disability risk score fell short of the best established clinical screening tests, such as mammography for breast cancer (detection rate 75% with a false positive rate of 8%)^32,33^ and faecal immunochemical test (FIT) for colon cancer (79%/6%)^34^, it was similar to those reported for widely used cardiovascular disease risk scores, such as QRISK2 (detection and false positive rates 40% and 13% for men and 26% and 6% for women)^12^ and thus appears to provide a useful tool to aid decisions of targeting preventive interventions. More specifically, our risk calculator had a relatively high false positive rate for test positive thresholds that allowed high detection rates. Conversely, the use of a threshold that provided low (approximately 5%) false positive rate, resulted in detection of only 20–25% of disability cases. This means that the score is useful in informing the targeting of interventions with no significant harm from overtreatment and in informing referrals to more detailed assessments for potential tailored preventive measures. By contrast, the score should not be used for expensive or new interventions with uncertain safety profiles as many people who will not benefit from the intervention will be targeted. The same applies to predictors of cardiovascular disease that are currently used in health care.

The present study benefits from the use of data which are from a country (Finland) where ascertainment of work disability pension was possible with linkage to comprehensive records from the national pension register with virtually full coverage for those in employment^10,11^. Large sample size, contributing to higher precision and lower risk of type 2 error, and the relatively high response rate are additional strengths. The age and sex distribution (mean age 43.1, 80% women) in the analytic sample corresponded to those in the eligible population of 133,966 municipal employees (mean age 45.7, 80% women)^13^. We defined disease groups using data from self-reported physician-diagnosed conditions. Validation studies supported the accuracy of self-reports as a measure of prevalent chronic diseases^35–44^.

This study has also several limitations. Although work disability is defined by impairment, receipt of a disability pension may additionally be dependent on non-medical factors, such as disability pension regulations, the work environment, the nature of the job, and the extent to which a workplace is prepared to accommodate the disability. Our study largely comprised women and all study participants were drawn from public sector workplaces, dominated by professionals, such as health care workers and teachers, deviating from the general population in which the incidence of work disability is greater^11^. However, among the Finnish workforce during the years 1997–2006, the yearly number of men and women with incident work disability was 111 and 100 per 10,000 persons^45^. This is higher but still comparable with the 89 per 1000 person-years in our total population. The generalizability of the present findings should therefore be investigated in different settings, study populations and other countries with different disability pension policies. Predictive performance may vary by country because in addition to the impairment, receipt of a disability pension is dependent on non-medical factors, such as disability pension regulations, the work environment, the nature of the job, and the extent to which the workplace is prepared to accommodate the disability^46^. Additionally, we followed the same parametric methodological approach as in the original FIOH study^2^, although a non-parametric approach (for example discrete-time models) would have avoided parametric assumptions.

## Conclusion

Detection of individuals at high risk is a precondition for effective targeted interventions to prevent long-term work disability and a basis for developing cost-effective strategies to avoid early labour-market exit. Predictive performance of the simple and cost-free FIOH-work disability risk score was comparable to those observed for established widely-used risk scores for other outcomes, such as cardiovascular diseases^47^. These findings suggest that it is possible to predict work disability in a working population with chronic disease using a scalable internet-based tool with a reasonable accuracy and thus aid decisions of targeted interventions and referrals to detailed assessments of tailored interventions.

## Supplementary Information

Supplementary Information.

## Data Availability

Statistical syntax for the analysis of the present study is available in Appendix, pp. 19–21. Pseudonymised questionnaire data from the FPS study can be shared upon request to Dr Jenni Ervasti (jenni.ervasti@ttl.fi). Linked health records require separate permission from the Findata, the Health and Social Data Permit Authority in Finland.
