# Supplementary material for: Predicting work disability among people with chronic conditions: a prospective cohort study
Source: Sci Rep. 2023 Apr 18;13:6334. doi: 10.1038/s41598-023-33120-3 (PMC10113323; doi:10.1038/s41598-023-33120-3)
Supplement: Supplementary file 1 — Supplementary Information. [file 41598_2023_33120_MOESM1_ESM.pdf]

Appendix

Contents Page

Table S1. Potential predictors 2
Table S2. Coefficients for formulating linear predictor in the original model 5
Table S3. Cause of work disability at follow-up in all participants and subgroups 6
Figure S1. Bivariate association between individual predictor items at baseline and work disability at follow-up in participants with musculoskeletal disorder at baseline 7
Figure S2. Bivariate association between individual predictor items at baseline and work disability at follow-up in participants with migraine at baseline 8
Figure S3. Bivariate association between individual predictor items at baseline and work disability at follow-up in participants with hypertension at baseline 9
Figure S4. Bivariate association between individual predictor items at baseline and work disability at follow-up in participants with respiratory disease at baseline 10
Figure S5. Bivariate association between individual predictor items at baseline and work disability at follow-up in participants with depression at baseline 11
Figure S6. Bivariate association between individual predictor items at baseline and work disability at follow-up in participants with diabetes at baseline 12
Figure S7. Bivariate association between individual predictor items at baseline and work disability at follow-up in participants with cancer at baseline 13
Figure S8. Bivariate association between individual predictor items at baseline and work disability at follow-up in participants with coronary heart disease at baseline 14
Figure S9. Bivariate association between individual predictor items at baseline and work disability at follow-up in participants with comorbid depression and cardiometabolic disease (diabetes, CHD or stroke) at baseline 15
Table S4. Coefficients for new predictive models 16
Table S5. Sensitivity, specificity and positive and negative predictive value for the original prediction model in all participants and subgroups of individuals with no history of sickness absence and those with a chronic condition at baseline 18
Statistical code 19

Table S1. Potential predictors

**Variable**
Age
Sex
Socioeconomic status
BMI
Height in cm
Weight in kg
Smoking
Do you smoke or have you smoked regularly (every day or almost every day)?
Alcohol consumption
Have you ever had at least a glass of an alcoholic beverage?
How many times a week you consume beer?
…wine?
…spirits?
How many times have you passed out from drinking during the past year?
Inactivity
During the past year, how many hours in a week have you walked?
…walked briskly?
…jogged?
…ran?
GHQ
In past weeks have you been able to concentrate?
...loss of sleep over worry
...playing a useful part
...capable of making decisions
...felt constantly under strain
...couldn't overcome difficulties
...able to enjoy day-to-day activities
...able to face problems
...feeling unhappy and depressed
...losing confidence
...thinking of self as worthless
...feeling reasonably happy
Number of chronic diseases
Allergic rhinitis
Asthma or chronic bronchitis
Hypertension
Diabetes
Myocardial infarction or angina pectoris
Cerebrovascular disorder (transient ischaemic attack)
Gastric or duodenal ulcer
Migraine
Depression
Other mental disorder

Cancer
Musculoskeletal disorder (i.e. arthrosis, rheumatoid arthritis or sciatica)
Self-rated health
Jenkins sleep scale

  How many times in the past 4 weeks have you had ...trouble falling a sleep
  …frequent awakenings during the night
  ...trouble remaining asleep
  ...feelings of fatigue and sleepiness despite receiving a typical night's rest

No. of sickness absences in previous year
Job strain
Job control

  My work requires creativity
  My work requires me to learn new things
  My work involves a lot of repetitive tasks
  I have a say in the tasks included in my work
  My work requires highly developed skills
  I have very little freedom to decide how I do my work

Job demand

  My work requires a lot of effort
  I am expected to do unreasonable amount of work
  I have sufficient time to get my work done

Relational justice

  Your supervisor considers your viewpoint
  Your supervisor is able to suppress personal biases
  Your supervisor provides you with timely feedback about the decision and its implications
  Your supervisor treats you with kindness and consideration
  Your supervisor shows concern for your rights as an employee
  Your supervisor takes steps to deal with you in a truthful manner

Procedural justice

  Procedures designed to… collect accurate information necessary for making decisions
  …provide opportunities to appeal or challenge the decision
  …have all sides affected by the decision represented.
  …generate standards so that decision could be made with consistency.
  …hear the concerns of all those affected by the decision.
  …provide useful feedback regarding the decision and its implementation
  …allow for requests for clarification or additional information about the decision.

Participatory safety

  People keep each other informed about work-related issues in the team
  There are real attempts to share information throughout the team
  We have a "we are in it together" attitude
  People feel understood and accepted by each other

Support for innovation

  People in this team are always searching for fresh, new ways of looking at problems
  In this team we take the time needed to develop new ideas
  People in the team co-operate in order to help develop and apply new ideas

Vision

To what extent do you think your team's objectives are clearly understood by other members of the team?
How far are you in agreement with these objectives?
To what extent do you think your team's objectives can actually be achieved?
How worthwhile do you think these objectives are?

Task orientation

Are team members prepared to question the basis of what the team is doing?
Does the team critically appraise potential weaknesses in what it is doing in order to achieve the best possible outcome?
Do members of the team build on each other's ideas in order to achieve the best possible outcome?

Social capital at work place

Do members of the team build on each other's ideas in order to achieve the best possible outcome?
People keep each other informed about work-related issues in the team
We have a "we are in it together" attitude
People feel understood and accepted by each other
People in the team co-operate in order to help develop and apply new ideas
?
Your supervisor treats you with kindness and consideration
Your supervisor shows concern for your rights as an employee
Your supervisor takes steps to deal with you in a truthful manner

Effort-Reward imbalance

Effort

How much of your skills and resources you invest in your work?

Reward

Do you feel that you get value for money for your work?
Do you feel that you get recognition and respect for your work?
Do you feel that you get personal satisfaction of your work?

Shift work

Night shift

Table S2. Coefficients for formulating linear predictor in the FIOH-model

Predictor	Beta coefficient
Age <35	0
Age=35-39	-0.2339
Age=40-44	-0.4356
Age=45-49	-0.8825
Age=50-54	-1.2873
Age=55+	-1.5418
BMI<18.5	-0.1724
BMI 18.5-24.9	0
BMI=25-29.9	-0.0668
BMI=30+	-0.1753
SEP=1	0
SEP=2	-0.0457
SEP=3	-0.3171
SEP=4	-0.3213
SEP=5	-0.546
SEP=6	-0.5294
SEP=7	-0.6597
Smoking=NO	0
Smoking=YES	-0.1638
Chronic illness=0	0
Chronic illness=1	-0.2252
Chronic illness=2	-0.4462
Chronic illness=3	-0.5342
Self-rated health=1	0
Self-rated health=2	-0.2348
Self-rated health=3	-0.5539
Self-rated health=4	-1.1336
Self-rated health=5	-1.5182
Difficulty falling asleep=1	0
Difficulty falling asleep=2	-0.0281
Difficulty falling asleep=3	-0.0769
Difficulty falling asleep=4	-0.1267
Difficulty falling asleep=5	-0.2014
Difficulty falling asleep=6	-0.2245
No. sickness absences in previous year=0	0
No. sickness absences in previous year=1	-0.4334
No. sickness absences in previous year=2	-0.7413
No. sickness absences in previous year=3	-1.133

Intercept = 5.7912

Scale = 1.2046

Table S3. Cause of work disability at follow-up in all participants and subgroups

			Disease group at baseline								
	All	Low-risk population*	MSD	Migraine	Hypertension	Respiratory disease	Depression	Diabetes	Cancer	CHD	DCD
	88521	73996	33601	22065	16793	15372	14347	3829	3584	1761	1162
Mean age (SD) at baseline	43.1 (9.8)	42.7 (9.9)	48.5 (8.4)	43.4 (9.7)	50.8 (7.5)	45.1 (9.8)	45.9 (9)	49.5 (9.3)	50.8 (8)	53.5 (7)	50.2 (8.4)
Mean follow-up time	8.6	8.8	7.5	8.4	7.1	7.9	7.8	6.8	6.7	5.9	6.3
N of work disability cases at follow-up	6836	4029	4734	2186	2539	1883	2392	628	509	398	322
Insidence per 1000 person-years	8.9	6.2	18.7	11.8	21.3	15.5	21.5	24.3	21.1	38.4	44.3
Cause of disability pension, N (%)											
ICD-10 M-codes (musculoskeletal disorders)	3021 (44.2)	1653 (41.0)	2607 (55.1)	982 (44.9)	1191 (46.9)	812 (43.1)	749 (31.3)	240 (38.2)	144 (28.3)	153 (38.4)	93 (28.9)
ICD-10 F-codes (mental and behavioural disorders)	1618 (23.7)	930 (23.1)	892 (18.8)	576 (26.3)	511 (20.1)	482 (25.6)	1049 (43.9)	114 (18.2)	82 (16.1)	67 (16.8)	116 (36)
ICD-10 C-codes (cancers)	472 (6.9)	331 (8.2)	243 (5.1)	118 (5.4)	124 (4.9)	97 (5.2)	111 (4.6)	28 (4.5)	193 (37.9)	9 (2.3)	10 (3.1)
ICD-10 G-codes (neurological disorders)	425 (6.2)	294 (7.3)	210 (4.4)	129 (5.9)	117 (4.6)	99 (5.3)	109 (4.6)	36 (5.7)	20 (3.9)	17 (4.3)	15 (4.7)
ICD-10 I-codes (cardiovascular disease)	398 (5.8)	259 (6.4)	229 (4.8)	123 (5.6)	225 (8.9)	103 (5.5)	102 (4.3)	76 (12.1)	19 (3.7)	102 (25.6)	36 (11.2)
Other codes	902 (13.2)	562 (13.9)	553 (11.7)	258 (11.8)	371 (14.6)	290 (15.4)	272 (11.4)	134 (21.3)	51 (10)	50 (12.6)	52 (16.1)

Abbreviations : CHD, coronary heart disease; DCD, comorbid depression and cardiometabolic disease (diabetes, CHD or stroke); MSD, musculoskeletal disorder

*Participants with no sickness absence during one year before baseline

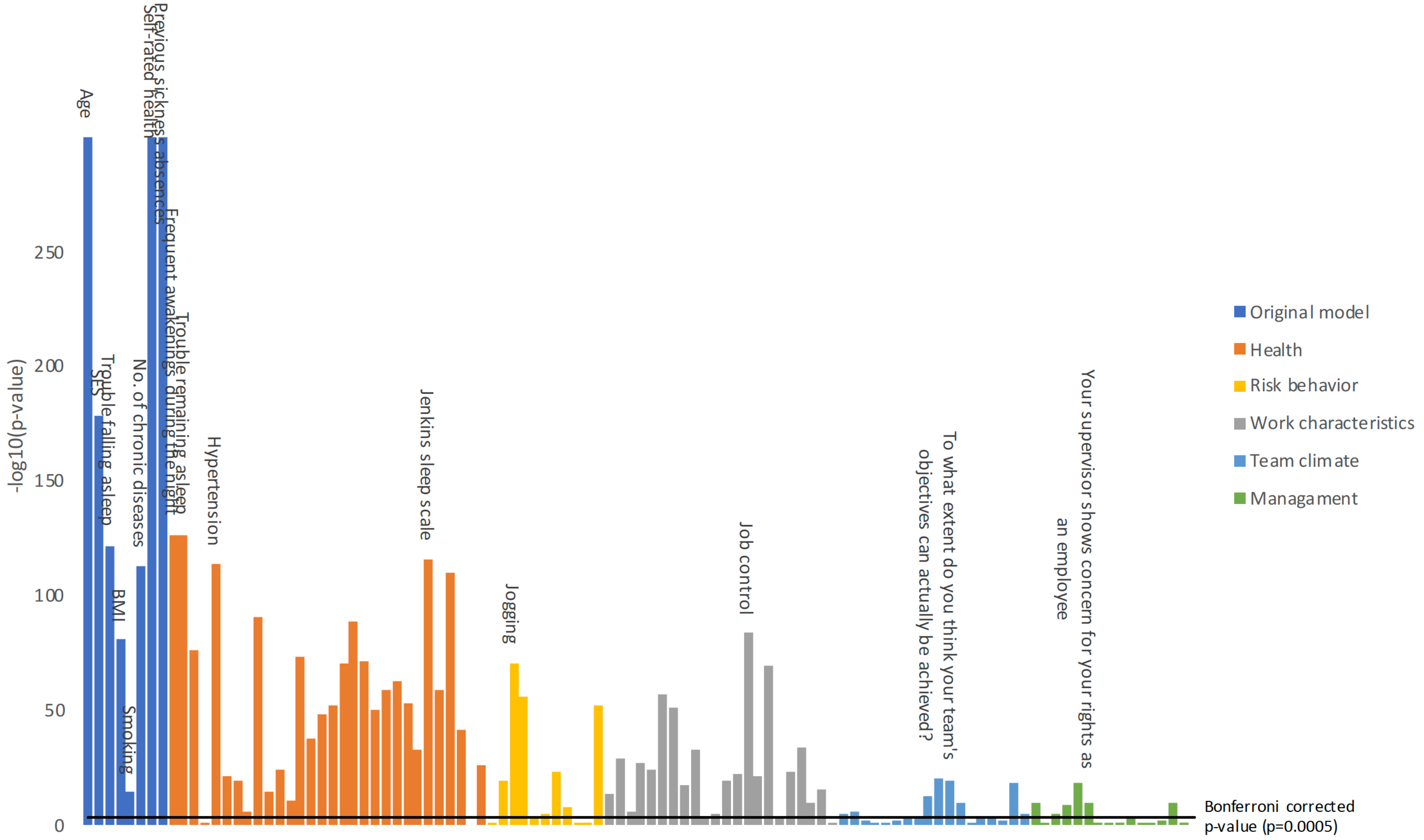

Figure S1. Bivariate association between individual predictor items at baseline and work disability at follow-up in participants with musculoskeletal disorder at baseline. Original model refers to the FIOH algorithm.

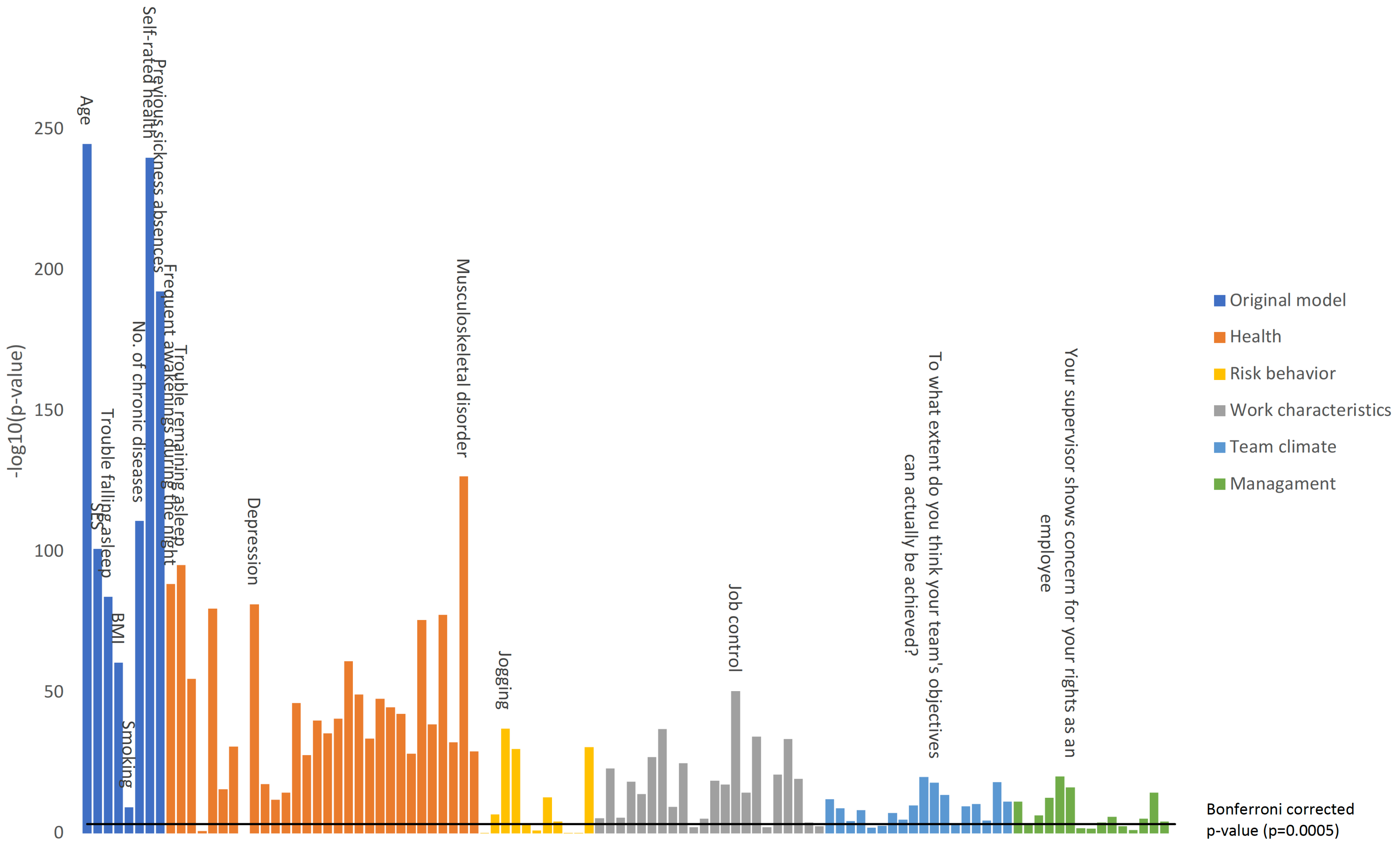

Figure S2. Bivariate association between individual predictor items at baseline and work disability at follow-up in participants with migraine at baseline. Original model refers to the FIOH algorithm.

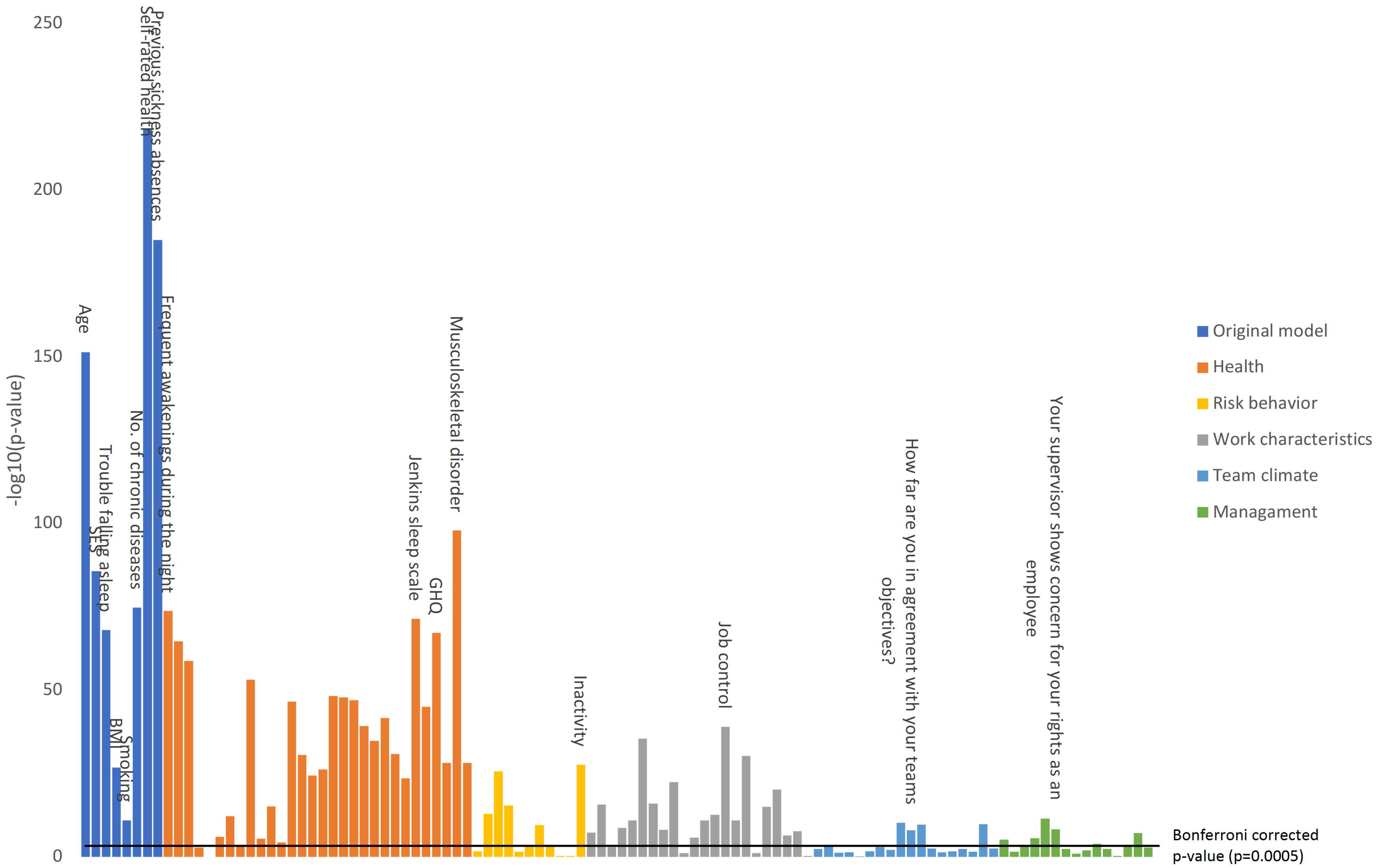

Figure S3. Bivariate association between individual predictor items at baseline and work disability at follow-up in participants with hypertension at baseline. Original model refers to the FIOH algorithm.

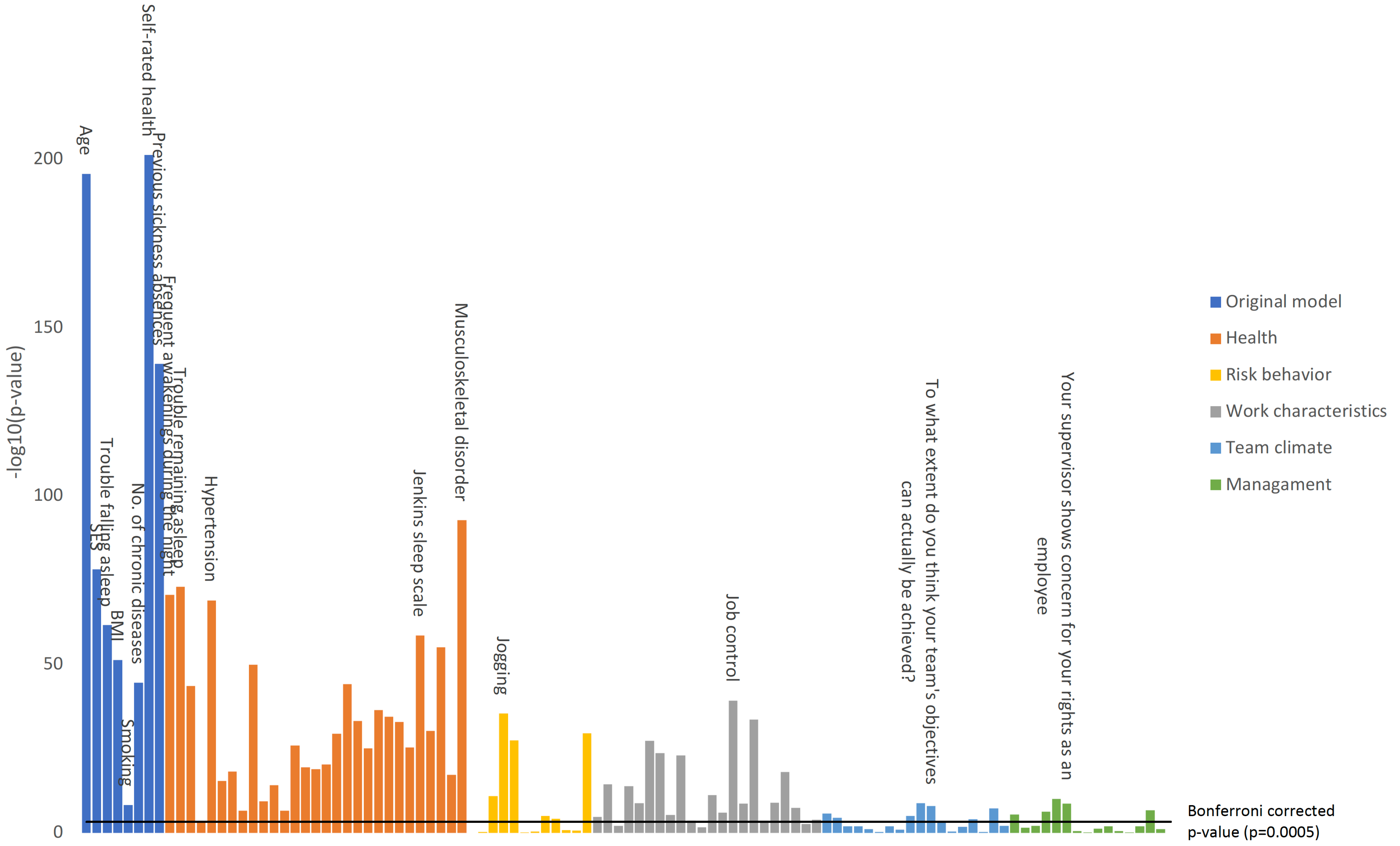

Figure S4. Bivariate association between individual predictor items at baseline and work disability at follow-up in participants with respiratory disease at baseline. Original model refers to the FIOH algorithm.

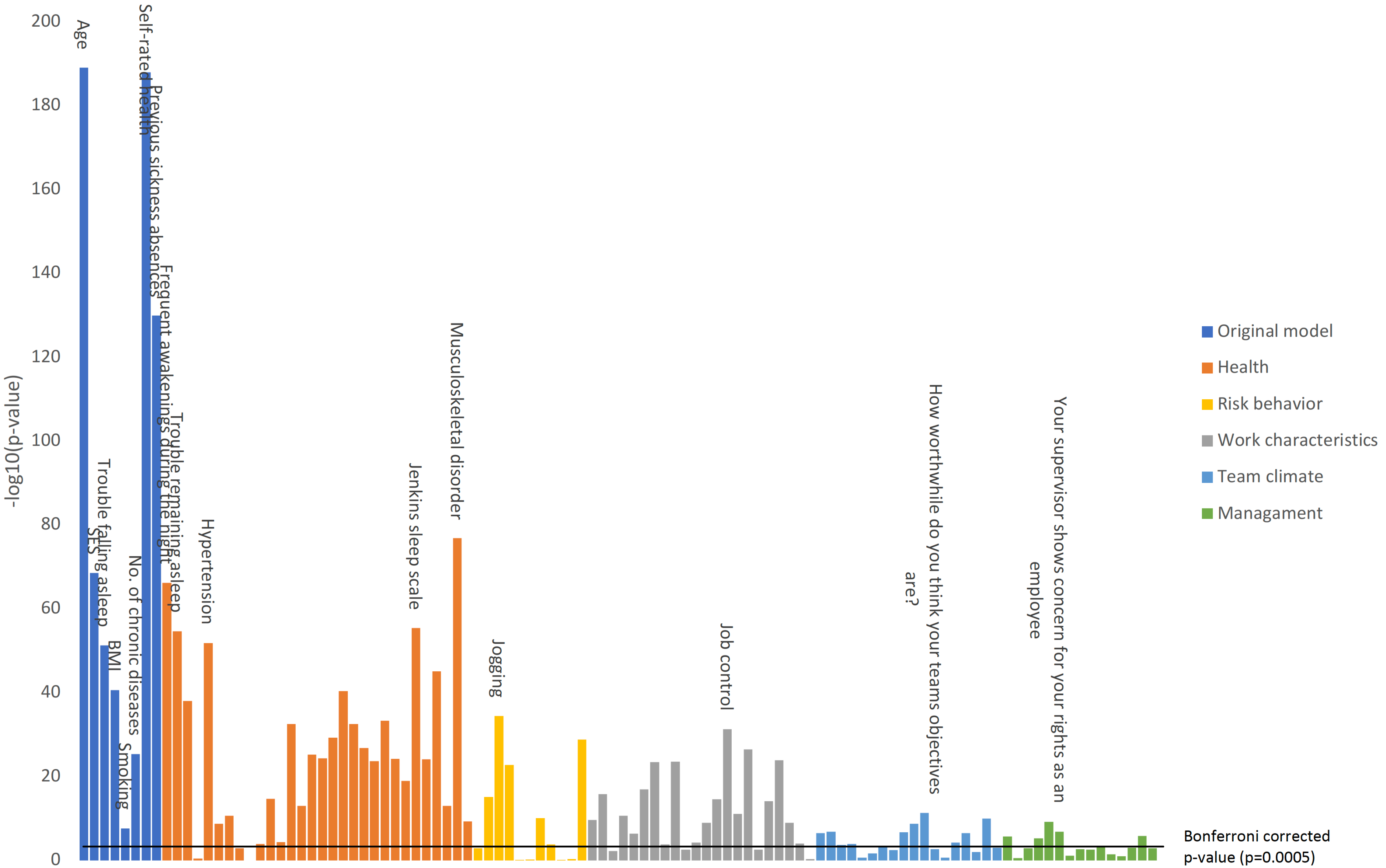

Figure S5. Bivariate association between individual predictor items at baseline and work disability at follow-up in participants with depression at baseline. Original model refers to the FIOH algorithm.

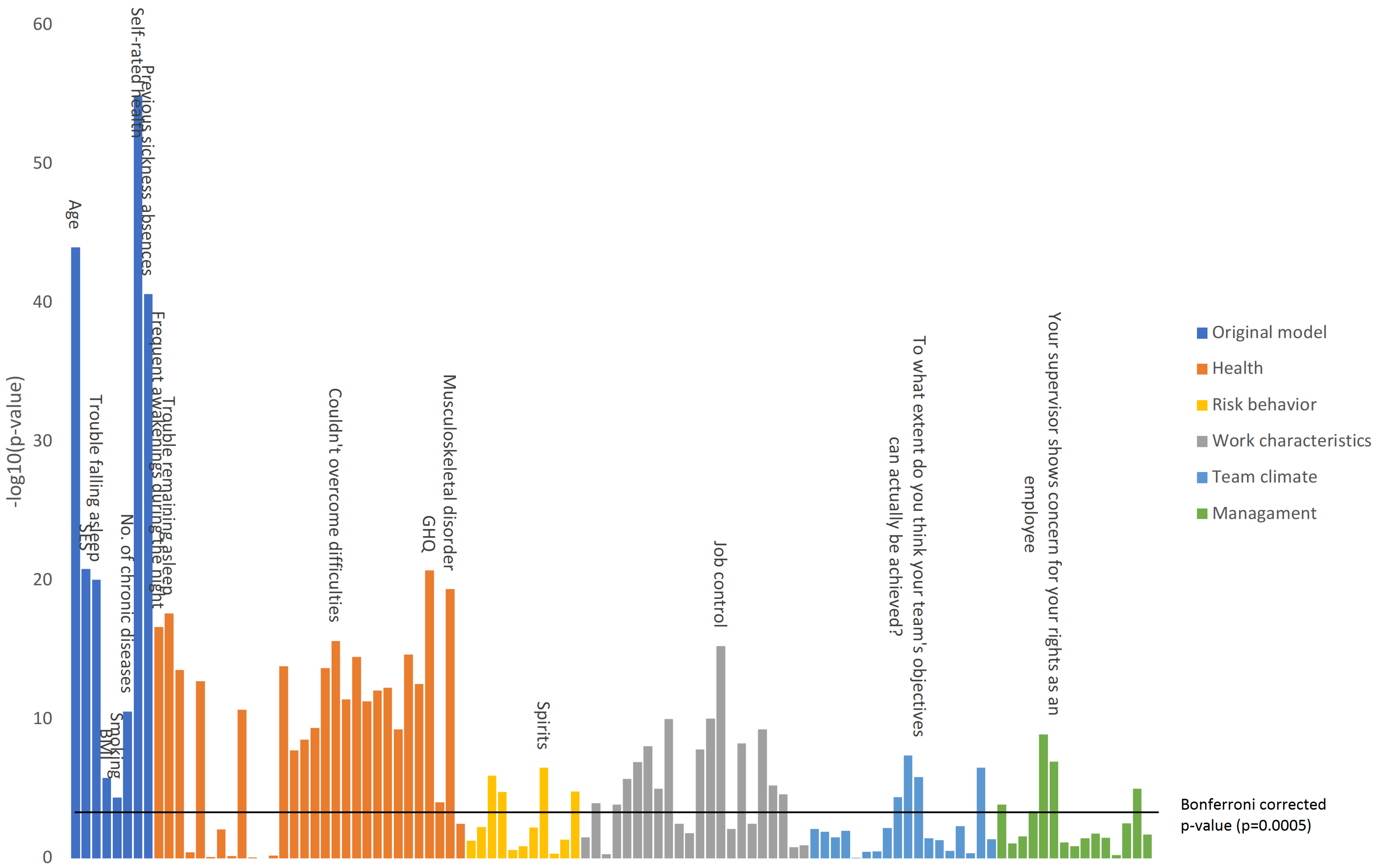

Figure S6. Bivariate association between individual predictor items at baseline and work disability at follow-up in participants with diabetes at baseline. Original model refers to the FIOH algorithm.

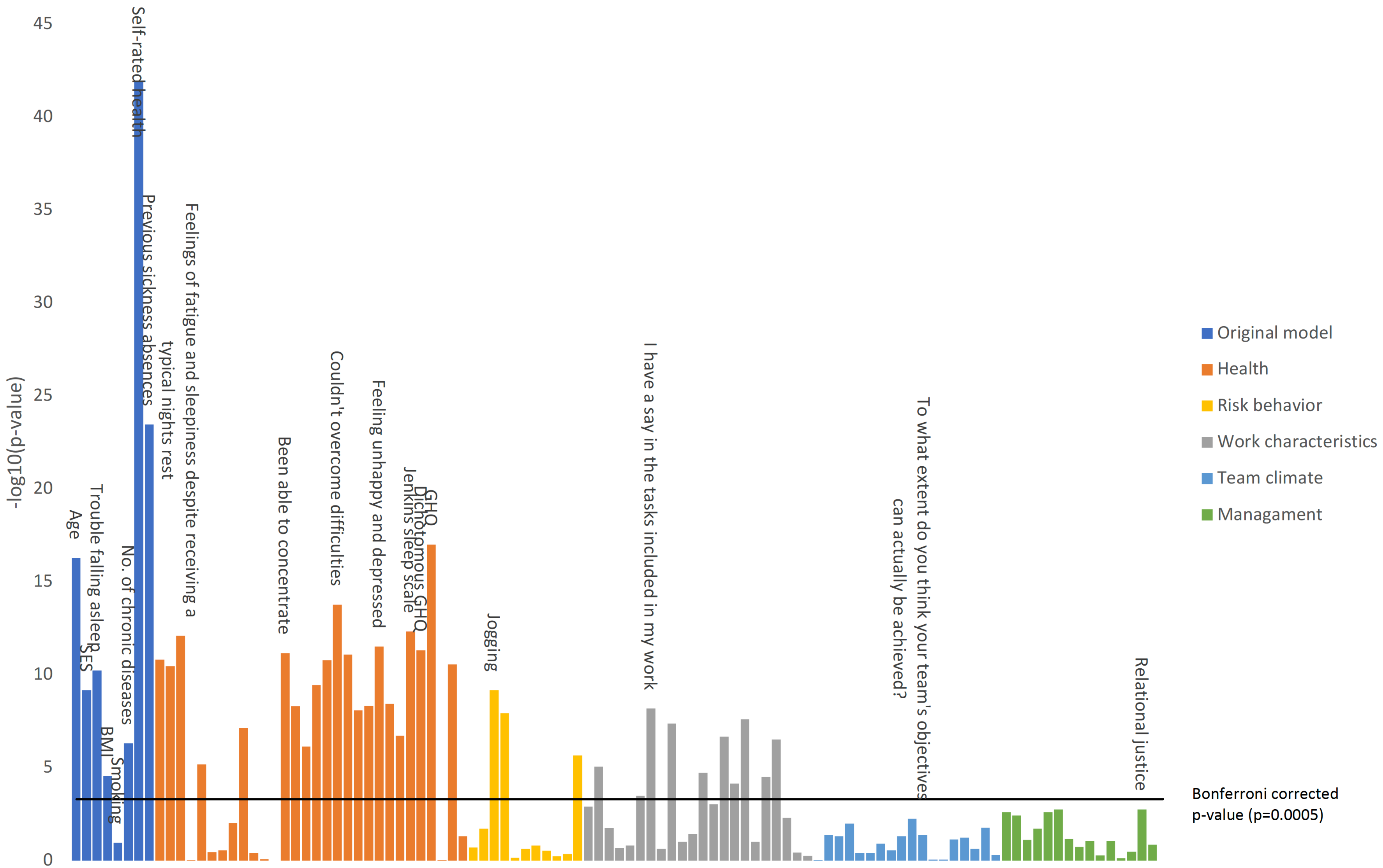

Figure S7. Bivariate association between individual predictor items at baseline and work disability at follow-up in participants with cancer at baseline. Original model refers to the FIOH algorithm.

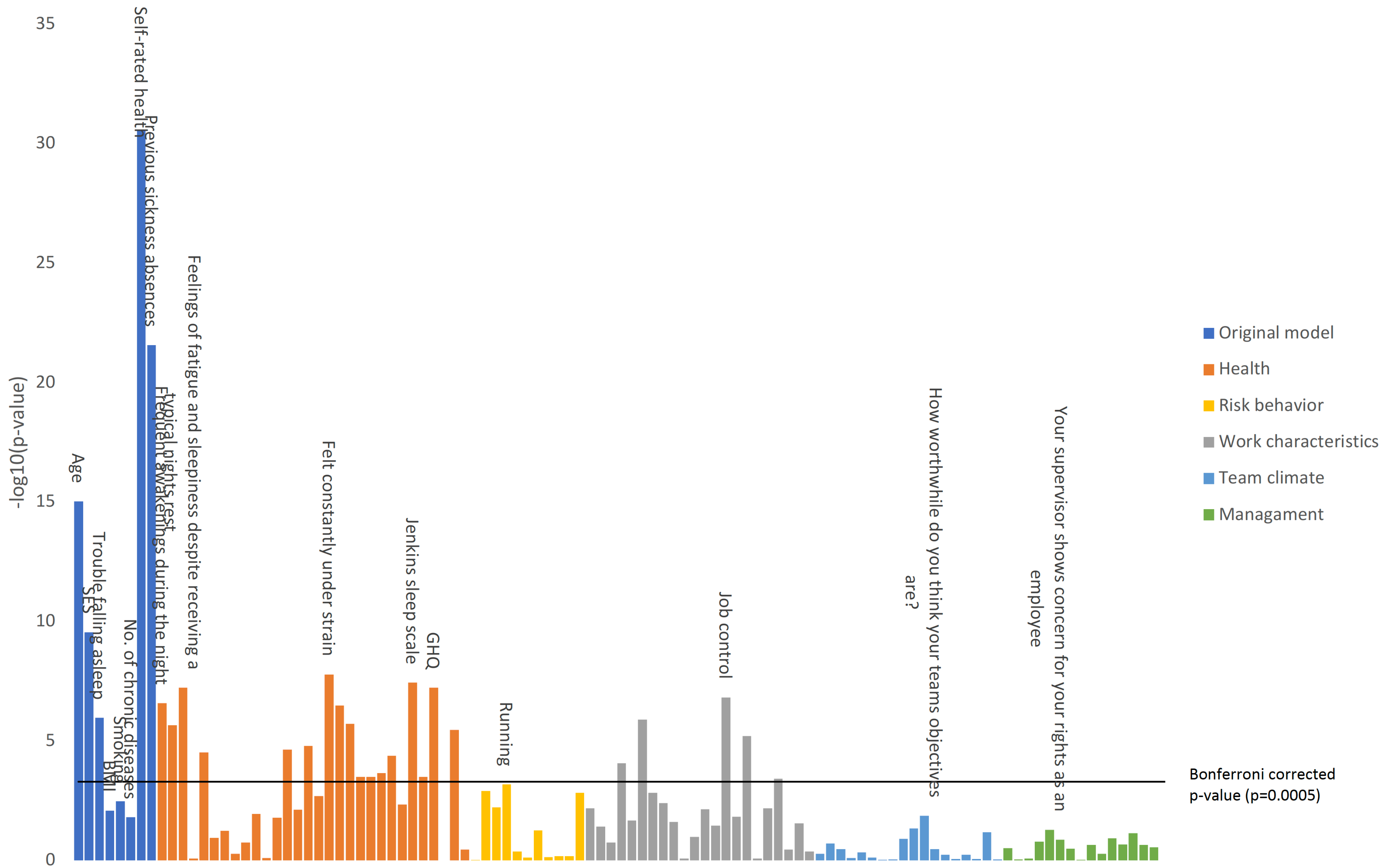

Figure S8. Bivariate association between individual predictor items at baseline and work disability at follow-up in participants with coronary heart disease at baseline. Original model refers to the FIOH algorithm.

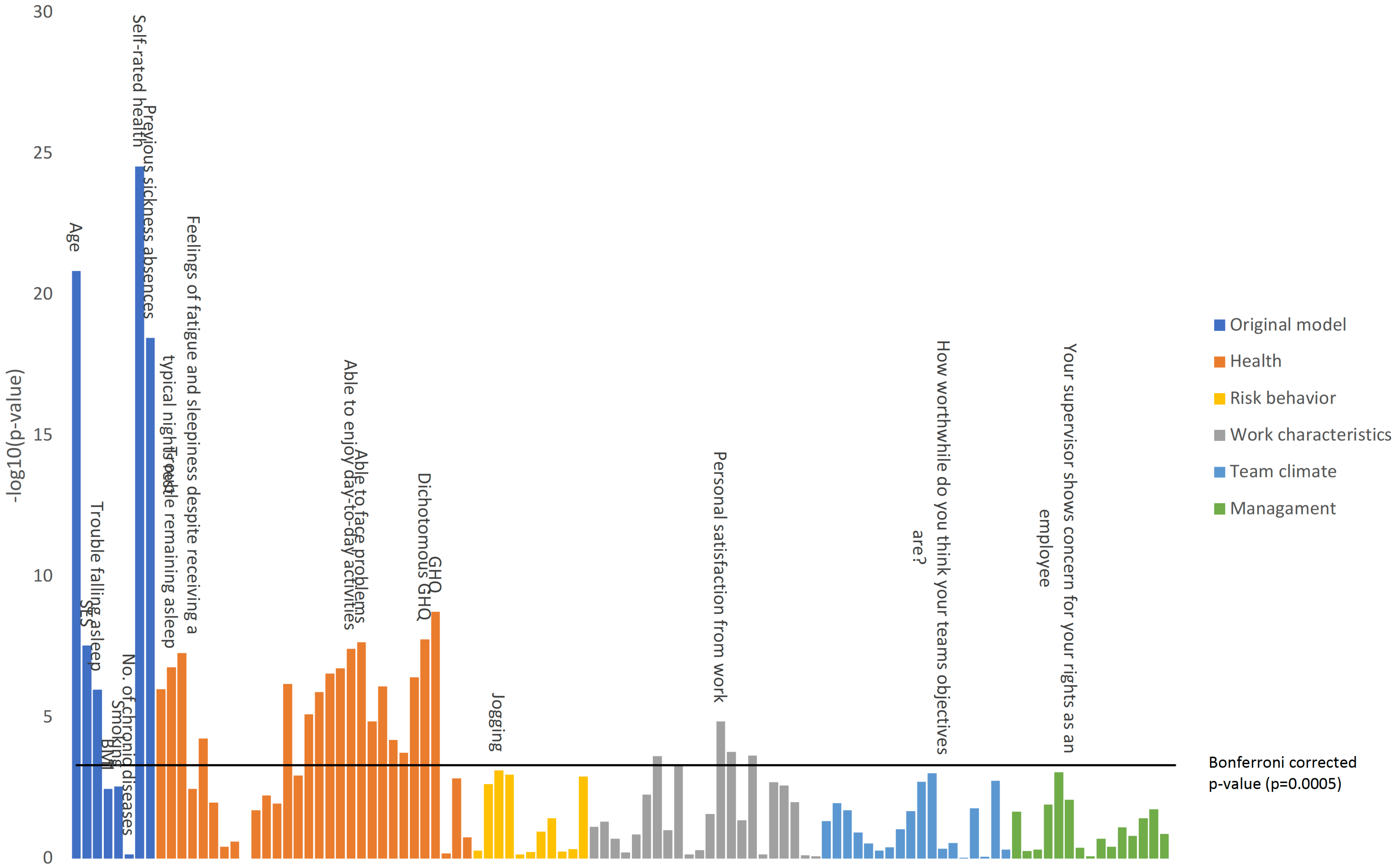

Figure S9. Bivariate association between individual predictor items at baseline and work disability at follow-up in participants with comorbid depression and cardiometabolic disease (diabetes, CHD or stroke) at baseline. Original model refers to the FIOH algorithm.

Table S4. Coefficients for new predictive models

			Coefficient/class							Legend
Disease	Variable	Classes	1	2	3	4	5	6	7	
**MSD, C=0.80 (0.79, 0.81)**										
	Age	1-6	0	-0.2873	-0.4692	-1.0077	-1.4558	-1.671		1='<35', 2='35-39', 3='40-44', 4='45-49', 5='50-54', 6='55+'
	BMI	1-4	-0.0542	0	-0.076	-0.1397				1='<18.5', 2='18.5-<25', 3='25-<30', 4='30+'
	SES	1-7	0	-0.043	-0.4308	-0.3418	-0.6404	-0.6046	-0.7126	1='Manager', 2=Senior specialist', 3='Specialist', 4='Office worker', 5='Service worker', 6='Process worker', 7='Other'
	Smoking	1-2	0	-0.124						1='No', 2='Yes'
	Self-rated health	1-5	0	-0.2817	-0.6463	-1.1033	-1.5051			1='Good', 2='Rather good', 3='Moderate', 4='Rather poor', 5='Poor'
	Number of sickness absences in previous year	1-4	0	-0.4247	-0.6184	-0.8252				1='0', 2='1', 3='2', 4='3+'
	Self-reported depression	1-2	0	-0.2389						1='No', 2='Yes'
	Effort*	1-5	0	0.0075	-0.1406	-0.1483	-0.2404			Likert scale ranging from 1 = "very little" to 5 = "very much"
	Intercept / Log(Scale)		6.0473 / -0.3264							
**Migraine, C=0.83 (0.83, 0.84)**										
	Age	1-6	0	-0.2667	-0.3559	-0.8367	-1.2783	-1.4647		1='<35', 2='35-39', 3='40-44', 4='45-49', 5='50-54', 6='55+'
	BMI	1-4	-0.0173	0	-0.0696	-0.153				1='<18.5', 2='18.5-<25', 3='25-<30', 4='30+'
	SES	1-7	0	0.0676	-0.2944	-0.2039	-0.4635	-0.3571	-0.5776	1='Manager', 2=Senior specialist', 3='Specialist', 4='Office worker', 5='Service worker', 6='Process worker', 7='Other'
	Smoking	1-2	0	-0.1423						1='No', 2='Yes'
	Number of chronic illness	1-3	0	-0.245	-0.38					1='1', 2='2', 3='3+'
	Self-rated health	1-5	0	-0.1242	-0.4803	-0.9516	-1.1781			1='Good', 2='Rather good', 3='Moderate', 4='Rather poor', 5='Poor'
	Number of sickness absences in previous year	1-4	0	-0.4321	-0.6274	-0.8612				1='0', 2='1', 3='2', 4='3+'
	Self-reported MSD	1-2	0	-0.2403						1='No', 2='Yes'
	Intercept / Log(Scale)		5.5881 / -0.3712							
**Hypertension, C=0.79 (0.79, 0.80)**										
	Age	1-6	0	-0.708	-0.7202	-1.1009	-1.5534	-1.7417		1='<35', 2='35-39', 3='40-44', 4='45-49', 5='50-54', 6='55+'
	SES	1-7	0	-0.0674	-0.3639	-0.273	-0.5827	-0.558	-0.7137	1='Manager', 2=Senior specialist', 3='Specialist', 4='Office worker', 5='Service worker', 6='Process worker', 7='Other'
	Number of chronic illness	1-4	0	-0.1912	-0.3115	-0.3957				1='0', 2='1', 3='2', 4='3+'
	Self-rated health	1-5	0	-0.1254	-0.4543	-0.985	-1.2665			1='Good', 2='Rather good', 3='Moderate', 4='Rather poor', 5='Poor'
	Number of sickness absences in previous year	1-4	0	-0.425	-0.6817	-0.734				1='0', 2='1', 3='2', 4='3+'
	Shiftwork	1-2	0	-0.0799						1='No', 2='Yes'
	Effort	1-5	0	-0.2776	-0.2748	-0.3743	-0.4252			Likert scale ranging from 1 = "very little" to 5 = "very much"
	Self-reported MSD	1-2	0	-0.2358						1='No', 2='Yes'
	Intercept / Log(Scale)		6.3076 / -0.3527							
**Respiratory disease, C=0.82 (0.81, 0.83)**										
	Age	1-6	0	-0.3198	-0.4333	-0.8849	-1.2447	-1.4719		1='<35', 2='35-39', 3='40-44', 4='45-49', 5='50-54', 6='55+'
	SES	1-7	0	-0.0195	-0.456	-0.4361	-0.5588	-0.5352	-0.7523	1='Manager', 2=Senior specialist', 3='Specialist', 4='Office worker', 5='Service worker', 6='Process worker', 7='Other'
	Number of chronic illness	1-4	0	-0.1302	-0.3148	-0.4203				1='0', 2='1', 3='2', 4='3+'
	Self-rated health	1-5	0	-0.3066	-0.6458	-1.121	-1.4591			1='Good', 2='Rather good', 3='Moderate', 4='Rather poor', 5='Poor'
	Number of sickness absences in previous year	1-4	0	-0.4279	-0.5175	-0.7633				1='0', 2='1', 3='2', 4='3+'
	Effort	1-5	0	-0.1467	-0.0632	-0.2482	-0.3408			Likert scale ranging from 1 = "very little" to 5 = "very much"
	Innovation / time for new ideas*	1-5	0	-0.0095	0.0142	0.1904	0.0914			Likert scale ranging from 1 = "completely agree" to 5 = "completely disagree"
	Self-reported MSD	1-2	0	-0.2174						1='No', 2='Yes'
	Intercept / Log(Scale)		6.0205 / -0.3435							
**Depression, C=0.78 (0.77, 0.78)**										
	Age	1-6	0	-0.0818	-0.2438	-0.7123	-1.0733	-1.3342		1='<35', 2='35-39', 3='40-44', 4='45-49', 5='50-54', 6='55+'
	BMI	1-4	-0.0536	0	-0.0831	-0.196				1='<18.5', 2='18.5-<25', 3='25-<30', 4='30+'
	SES	1-7	0	0.0458	-0.2559	-0.2574	-0.4078	-0.3966	-0.5348	1='Manager', 2=Senior specialist', 3='Specialist', 4='Office worker', 5='Service worker', 6='Process worker', 7='Other'
	Number of chronic illness	1-3	0	-0.0697	-0.2303					1='1', 2='2', 3='3+'
	Self-rated health	1-5	0	-0.1492	-0.471	-0.9069	-1.2568			1='Good', 2='Rather good', 3='Moderate', 4='Rather poor', 5='Poor'
	Difficulty falling asleep	1-6	0	-0.043	-0.0966	-0.1399	-0.0956	-0.2353		1='Never', 2='1-3 nights/month', 3='once/week', 4='2-4 nights per week'', 5='5-6 nights per week', 6='Almost every night'
	Number of sickness absences in previous year	1-4	0	-0.3488	-0.5904	-0.9371				1='0', 2='1', 3='2', 4='3+'
	Self-reported MSD	1-2	0	-0.151						1='No', 2='Yes'
	Intercept / Log(Scale)		5.3043 / -0.2333							

Outcome	Variable	Range	1	2	3	4	5	6	7	Coding
**Diabetes, C=0.79 (0.77, 0.81)**										
	Age	1-6	0	-0.4991	-0.622	-0.9117	-1.4321	-1.4612		1='<35', 2='35-39', 3='40-44', 4='45-49', 5='50-54', 6='55+'
	SES	1-7	0	0.0428	-0.245	-0.1714	-0.3837	-0.4702	-0.5859	1='Manager', 2=Senior specialist', 3='Specialist', 4='Office worker', 5='Service worker', 6='Process worker', 7='Other'
	Self-rated health	1-5	0	-0.4466	-0.8065	-1.3214	-1.4737			1='Good', 2='Rather good', 3='Moderate', 4='Rather poor', 5='Poor'
	Number of sickness absences in previous year	1-4	0	-0.4449	-0.7388	-0.6014				1='0', 2='1', 3='2', 4='3+'
	Reward / personal satisfaction	1-5	0	0.1448	0.0096	-0.1635	-0.1707			Likert scale ranging from 1 = "very much" to 5 = "very little"
	Innovation / co-operation for new ideas*	1-5	0	-0.2719	-0.1255	0.0177	0.0754			Likert scale ranging from 1 = "completely agree" to 5 = "completely disagree"
	Self-reported depression	1-2	0	-0.2912						1='No', 2='Yes'
	Self-reported MSD	1-2	0	-0.1576						1='No', 2='Yes'
	Intercept / Log(Scale)		5.8554 / -0.2743							
**Cancer, C=0.74 (0.72, 0.76)**										
	Age	1-6	0	0.7137	-0.2989	-0.2182	-0.5742	-0.7943		1='<35', 2='35-39', 3='40-44', 4='45-49', 5='50-54', 6='55+'
	SES	1-7	0	0.0786	0.0184	0.0565	-0.2462	-0.0411	-0.2568	1='Manager', 2=Senior specialist', 3='Specialist', 4='Office worker', 5='Service worker', 6='Process worker', 7='Other'
	Self-rated health	1-5	0	-0.464	-0.7688	-1.388	-2.1199			1='Good', 2='Rather good', 3='Moderate', 4='Rather poor', 5='Poor'
	Number of sickness absences in previous year	1-4	0	-0.4614	-0.5213	-0.9575				1='0', 2='1', 3='2', 4='3+'
	Task orientation / weaknesses		0	0.2875	0.3744	0.4419	0.4931			Likert scale ranging from 1 = "very much" to 5 = "very little"
	Job control / a lot of say	1-5	0	-0.0219	-0.207	-0.2906	-0.3205			Likert scale ranging from 1 = "completely agree" to 5 = "completely disagree"
	Job control / little freedom	1-5	0	-0.0248	0.122	0.1797	0.3082			Likert scale ranging from 1 = "completely agree" to 5 = "completely disagree"
	Self-reported MSD	1-2	0	-0.2083						1='No', 2='Yes'
	Intercept / Log(Scale)		4.7732 / -0.1273							
**CHD, C=0.77 (0.74, 0.79)**										
	Age	1-6	0	0.8087	0.7104	-0.4283	-0.9428	-0.9543		1='<35', 2='35-39', 3='40-44', 4='45-49', 5='50-54', 6='55+'
	SES	1-7	0	0.318	0.0684	-0.011	-0.0548	-0.1018	-0.367	1='Manager', 2=Senior specialist', 3='Specialist', 4='Office worker', 5='Service worker', 6='Process worker', 7='Other'
	Number of chronic illness	1-3	0	-0.1758	-0.2125					1='1', 2='2', 3='3+'
	Self-rated health	1-5	0	-0.8038	-1.0533	-1.5925	-1.8411			1='Good', 2='Rather good', 3='Moderate', 4='Rather poor', 5='Poor'
	Number of sickness absences in previous year	1-4	0	-0.3633	-0.7461	-0.5799				1='0', 2='1', 3='2', 4='3+'
	Self-reported hypertension	1-2	0	-0.202						1='No', 2='Yes'
	Self-reported MSD	1-2	0	-0.2329						1='No', 2='Yes'
	Self-reported respiratory disease	1-2	0	0.1749						1='No', 2='Yes'
	Intercept / Log(Scale)		5.4092 / -0.2257							
**DCD, C=0.78 (0.75, 0.80)**										
	Age	1-6	0	-0.0598	-0.1713	-0.4495	-1.0428	-1.1964		1='<35', 2='35-39', 3='40-44', 4='45-49', 5='50-54', 6='55+'
	SES	1-7	0	0.4618	0.2905	0.4255	0.1116	0.2913	-0.0872	1='Manager', 2=Senior specialist', 3='Specialist', 4='Office worker', 5='Service worker', 6='Process worker', 7='Other'
	Smoking	1-2	0	-0.2495						1='No', 2='Yes'
	Self-rated health	1-5	0	0.0365	-0.566	-1.1546	-1.3794			1='Good', 2='Rather good', 3='Moderate', 4='Rather poor', 5='Poor'
	Number of sickness absences in previous year	1-4	0	-0.6047	-0.7199	-0.9259				1='0', 2='1', 3='2', 4='3+'
	Self-reported hypertension	1-2	0	-0.2578						1='No', 2='Yes'
	Innovation /problems*	1-5	0	0.1225	0.0802	0.3537	0.4378			Likert scale ranging from 1 = "completely agree" to 5 = "completely disagree"
	Self-reported allergic rhinitis	1-2	0	0.1979						1='No', 2='Yes'
	Intercept / Log(Scale)		4.4099 / -0.1529							

Effort: "How much do you feel you invest in your job in terms of skill and energy?"
Innovation / time for new ideas: "In this team we take the time needed to develop new ideas"
Reward / personal satisfaction: "Do you feel that you get personal satisfaction of your work?"
Innovation / co-operation for new ideas: 'People in the team co-operate in order to help develop and apply new ideas'
Innovation / problems: 'People in this team are always searching for fresh, new ways of looking at problems'
Job control / a lot of say: 'I have a lot of say in the tasks included in my work'
Job control / little freedom: 'I have very little freedom to decide how I do my work'
Task orientation / weaknesses: 'Does the team critically appraise potential weaknesses in what it is doing in order to achieve the best possible outcome?'

Table S5. Sensitivity, specificity and positive and negative predictive value for the FIOH-model in all participants and subgroups of individuals with no history of sickness absence and those with a chronic condition at baseline

Predictive performance for a positive test	Cut-off (%) for a positive test result						
	5	10	20	30	40	50	60
**Musculoskeletal disorders**							
Sensitivity	95.3	86.6	65.9	46.6	32.7	21.1	12.8
Specificity	29.8	49.6	73.4	86.4	92.9	96.4	98.3
Positive predictive value	18.2	22.0	28.8	36.0	43.2	48.9	54.6
Negative predictive value	97.5	95.8	92.9	90.8	89.4	88.2	87.3
**Migraine**							
Sensitivity	92.0	83.1	63.6	47.6	33.3	22.1	14.3
Specificity	45.8	63.0	80.7	90.0	94.8	97.4	98.8
Positive predictive value	15.7	19.8	26.6	34.5	41.4	48.5	57.3
Negative predictive value	98.1	97.1	95.3	94.0	92.8	91.9	91.3
**Hypertension**							
Sensitivity	96.4	89.6	71.2	53.2	37.3	24.8	15.2
Specificity	19.5	38.3	65.6	82.1	90.6	95.1	97.8
Positive predictive value	17.6	20.6	26.9	34.6	41.5	47.5	55.0
Negative predictive value	96.8	95.4	92.8	90.8	89.0	87.7	86.6
**Respiratory disease**							
Sensitivity	93.0	84.8	66.0	49.9	35.8	24.1	15.1
Specificity	40.1	57.1	76.8	87.1	92.8	96.2	98.2
Positive predictive value	17.8	21.6	28.4	35.0	41.2	47.1	54.2
Negative predictive value	97.6	96.4	94.2	92.6	91.2	90.1	89.2
**Depression**							
Sensitivity	93.4	86.2	69.7	53.7	40.1	27.4	18.0
Specificity	27.7	45.7	68.3	81.5	89.3	93.9	97.0
Positive predictive value	20.5	24.1	30.6	36.7	42.9	47.3	54.3
Negative predictive value	95.4	94.3	91.8	89.8	88.2	86.6	85.5
**Diabetes**							
Sensitivity	97.0	91.6	77.7	62.4	46.3	32.8	20.7
Specificity	19.2	32.0	53.7	72.4	83.9	91.4	95.7
Positive predictive value	19.1	20.9	24.8	30.7	36.1	42.8	48.7
Negative predictive value	97.0	95.1	92.5	90.8	88.8	87.4	86.0
**Cancer**							
Sensitivity	93.1	80.4	58.3	42.2	30.3	18.9	13.2
Specificity	21.2	40.7	66.5	81.2	90.3	95.7	97.9
Positive predictive value	16.4	18.3	22.4	27.1	34.1	42.3	51.1
Negative predictive value	94.9	92.6	90.6	89.5	88.7	87.7	87.2
**Coronary heart disease**							
Sensitivity	99.2	97.2	88.9	76.1	62.8	47.2	31.7
Specificity	5.6	12.5	33.6	54.5	69.7	81.0	89.7
Positive predictive value	23.5	24.5	28.1	32.8	37.7	42.1	47.4
Negative predictive value	96.3	94.0	91.2	88.7	86.5	84.0	81.8
**Comorbid depression and cardiometabolic disease**							
Sensitivity	98.1	96.3	90.7	79.8	67.7	52.8	38.5
Specificity	11.0	20.1	38.1	54.8	67.7	79.9	88.2
Positive predictive value	29.7	31.6	36.0	40.3	44.6	50.1	55.6
Negative predictive value	93.9	93.4	91.4	87.6	84.5	81.5	78.9
**Low-risk population**							
Sensitivity	81.6	63.5	35.0	17.2	8.3	3.5	1.2
Specificity	61.8	78.5	92.3	97.3	99.1	99.6	99.9
Positive predictive value	11.0	14.5	20.8	26.8	33.8	34.9	38.6
Negative predictive value	98.3	97.4	96.1	95.3	94.9	94.7	94.6
**Total cohort**							
Sensitivity	87.9	75.0	52.5	35.9	24.0	15.0	9.1
Specificity	56.3	73.0	88.0	94.3	97.2	98.6	99.4
Positive predictive value	14.4	18.9	26.7	34.6	41.9	48.0	55.3
Negative predictive value	98.2	97.2	95.7	94.6	93.9	93.3	92.9

Statistical code

Imputation of missing data

```
imputed <- mice(idata, m=1, method = "pmm", seed=1)
idata_all <-complete(imputed,1)
```

C-index

```
#Create survival object
sv <- Surv(data$tkseuraika, data$tkstatus)

#Calculate c-index
c_old <- rcorr.cens(data$predicts_old, sv)
old_lci <- round(c_old["C Index"]-1.96*(c_old["S.D."]/2),4)
old_hci <- round(c_old["C Index"]+1.96*(c_old["S.D."]/2),4)
```

Re-estimating the coefficients of the original model

```
#Define the model
formodel <- as.formula("Surv(tkseuraika, tkstatus) ~ as.factor(agecat)+
                        as.factor(bmi00)+
                        as.factor(ses)+
                        as.factor(smoking)+
                        as.factor(illnesses)+
                        as.factor(srh00)+
                        as.factor(sleep0)+
                        as.factor(sickabs)")
#Estimate new model
newmodel <- psm(formodel, data=data, dist="lognormal", x=T, y=T)
newmodel
```

An example of selecting a completely new set of predictors

```
#Create a formula vector to be used in redundancy analysis
forred <- as.formula(paste("~",paste0(allvars,collapse="+")))
forred
#Redundancy analysis
red <- redun(forred, nk=0, data=idata)
red

#Delete redundant variables from "allvars" and create a new vector called "vars_subset"
#that is used from here on out.
vars_subset <- allvars

for(d in 1:length(red$Out)){
  vars_subset <- vars_subset[vars_subset !=red$Out[d]]
```

```
}
vars_subset


######### Predictor selection (stepwise backward elimination) ###########

#First we fit a full parametric survival model
  #Define the model
  forwei <- as.formula(paste("Surv(", "tkseuraika",",","tkstatus",") ~", paste(vars_subset,
collapse='+')))
  #Run the analysis
  fullWei <- psm(forwei, data=idata, dist = "weibull")
fullWei
#Add linear predictor values from full Weibull model to dataframe
idata$weilp <- predict(fullWei, type="lp")

#Then an OLS model with the outcome from Weibull model as dependent variable
  #Define the model
  forols <- as.formula(paste("weilp ~", paste0(vars_subset, collapse='+')))
  #Run the predictor selection
  model = regsubsets(forols, data = idata,  method = "backward", nvmax=50)
  #Save summary of the results to "modelsum" (used to extract predictor names)
  modelsum <-summary(model)

nvars <- 8

#Extract variable names from "modelsum"
i<-2
iv<-vector()
modelnames <- colnames(modelsum$which[])
while(i<length(modelsum$which[nvars,])+1){
  if(modelsum$which[nvars,i]==1)
     iv <- c(iv,paste(modelnames[i]))
    i<-i+1
     }

##Define a formula from extracted variables
finalmodel <- as.formula(paste("Surv(tkseuraika, tkstatus) ~",paste(iv, collapse =  "+")))
finalmodel

##Add predicted values to dataframe from the model derived from backward elimination
weimodel<-psm(finalmodel, data=idata, dist="weibull", x=T, y=T)
weimodel
idata$predicts <- predict(weimodel, type="lp")


idata$agecat <- as.numeric(cut(idata$age,c(15,35,40,45,50,55,70), right = F))
```

```
idata$bmicat  <- as.numeric(cut(idata$bmi00,c(15,18.5,25,30,55), right = F))

idata$ sickabs_cat <- ifelse(idata$ sickabs>3,3,idata$ sickabs)

finalmodel <- as.formula(paste("Surv(tkseuraika, tkstatus) ~ as.factor(agecat) +
as.factor(K31_2) +
                          as.factor(ses) + as.factor(sickabs_cat) + as.factor(eff00) +
as.factor(tautisum00) +
                         as.factor(ttila00) + as.factor(k8y8_9_11) "))


##Add predicted values to dataframe from the model derived from backward elimination
weimodel<-psm(finalmodel, data=idata, dist="weibull", x=T, y=T)
weimodel
idata$predicts <- predict(weimodel, type="lp")
```
